# Chromatin Priming Renders T Cell Tolerance-Associated Genes Sensitive to Activation below the Signaling Threshold for Immune Response Genes

**DOI:** 10.1016/j.celrep.2020.107748

**Published:** 2020-06-09

**Authors:** Sarah L. Bevington, Sky T.H. Ng, Graham J. Britton, Peter Keane, David C. Wraith, Peter N. Cockerill

**Affiliations:** 1 Institute of Cancer and Genomic Sciences, College of Medical and Dental Sciences, University of Birmingham, Birmingham B15 2TT, UK; 2 Institute of Immunology and Immunotherapy, College of Medical and Dental Sciences, University of Birmingham, Birmingham B15 2TT, UK; 3 Precision Immunology Institute and Icahn Institute for Data Science and Genomic Technology, Icahn School of Medicine at Mount Sinai, New York, NY 10029, USA

**Keywords:** T cell, tolerance, chromatin, epigenetic, gene regulation, signaling, transcription factor, CTLA4, IL-10, Cbl-b

## Abstract

Immunological homeostasis in T cells is maintained by a tightly regulated signaling and transcriptional network. Full engagement of effector T cells occurs only when signaling exceeds a critical threshold that enables induction of immune response genes carrying an epigenetic memory of prior activation. Here we investigate the underlying mechanisms causing the suppression of normal immune responses when T cells are rendered anergic by tolerance induction. By performing an integrated analysis of signaling, epigenetic modifications, and gene expression, we demonstrate that immunological tolerance is established when both signaling to and chromatin priming of immune response genes are weakened. In parallel, chromatin priming of immune-repressive genes becomes boosted, rendering them sensitive to low levels of signaling below the threshold needed to activate immune response genes. Our study reveals how repeated exposure to antigens causes an altered epigenetic state leading to T cell anergy and tolerance, representing a basis for treating auto-immune diseases.

## Highlights

• Activation of immune response genes is suppressed in tolerant T cells
• Epigenetic priming of repressive genes is boosted when tolerance is established
• Inhibitory receptor genes have a lower threshold of activation in tolerant cells
• Induction of tolerance by peptides points toward a therapy for multiple sclerosis

## Graphical Abstract

**Figure:**
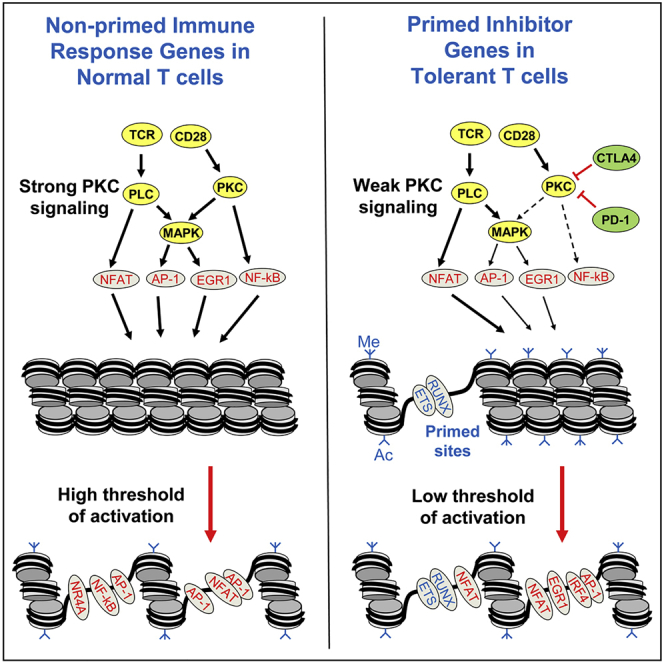

## Teaser

Autoimmune diseases such as multiple sclerosis (MS) are caused by T cells reacting to host proteins. Bevington et al. use escalating doses of a MS auto-antigen to induce tolerance in T cells in mice, and define a mechanism whereby antigen activates repressive genes while silencing immune response genes.

## Introduction

The maintenance of immunological homeostasis requires T cell responses to antigen (Ag) to be both tightly regulated and receptive to negative feedback. The engagement of effector T cells is dependent on the activation of multiple parallel signaling pathways, thereby ensuring appropriate immune responses. The magnitude of the response to T cell receptor (TCR) signaling can also be suppressed by inducing an anergic state in response to: (1) repeated exposure to Ag, resulting in tolerance (Sabatos-Peyton et al., 2010); (2) lack of co-receptor stimulation (Schwartz, 2003); or (3) negative feedback from inhibitory receptors, such as PD-1, CTLA4, LAG3, TIM3, and TIGIT, as seen in tumor-infiltrating lymphocytes (TILs) and exhausted T cells facing chronic exposure to viral Ags (Anderson et al., 2016, Wherry et al., 2007, Wherry and Kurachi, 2015). PD-1 reduces the strength of TCR signaling below the threshold for many immune response genes, but not for a subset of other inducible genes that have a lower activation threshold (Shimizu et al., 2020). However, the molecular basis for this phenomenon is unknown.

**Figure 1:**
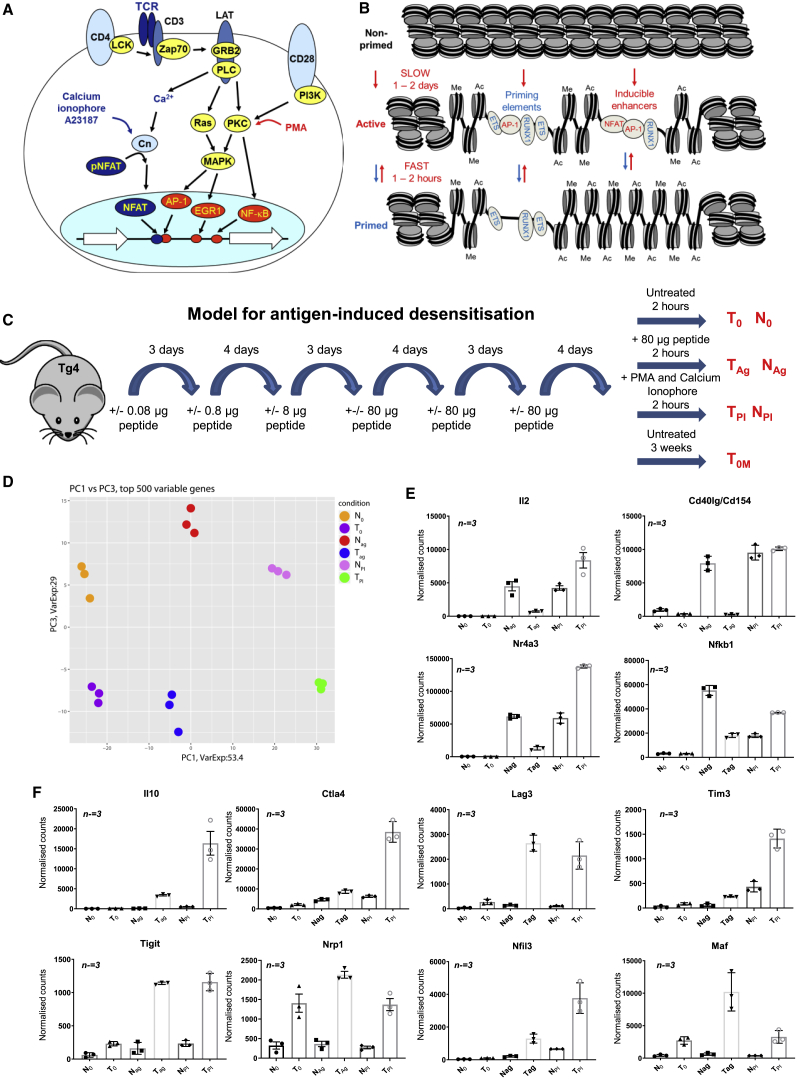
Gene Expression Analyses Comparing Tolerized T Cells with Naive T Cells (A) Model of the activation of immune response genes by TCR and CD28 signaling to inducible TFs. Calcium ionophore A23187 and PMA act on the same signaling pathways downstream of PLC. (B) The transcription factors activated by signaling (NFAT, AP-1) induce chromatin remodeling at immune response genes at both priming elements and inducible enhancers. Enhancer activation initiates transcription of their target genes. When signaling ceases, primed DHSs are stably maintained as regions of methylated (me) and acetylated (ac) chromatin by the retention of constitutively expressed transcription factors (ETS, RUNX). (C) Schematic showing the dose escalation protocol used in tolerizing Tg4 mice by subcutaneous injections with MBP Ac1-9-specific TCR. (D) Principle-component analysis of the RNA-seq datasets for the top 500 most variable genes for N_0_, T_0_, N_Ag_, T_Ag_, N_PI_, and T_PI_. RNA-seq data were taken from three biological replicates. (E and F) Normalized average RNA-seq counts for classical immune response (E) and immune-modulatory genes (F). Error bars represent the standard deviation from three biological replicates.

During a normal immune response, the co-activation of TCR and CD28 signaling in effector T cells leads to the transcriptional activation of hundreds of genes via inducible transcription factors (TFs), such as NFAT, AP-1, nuclear factor κB (NF-κB), and EGR1 (Yukawa et al., 2020; Figure 1A). These same TFs are also strongly induced by the phorbol ester phorbol myristate acetate (PMA) and the calcium ionophore A23187 (PI), which cooperate by activating diverging signaling pathways downstream of phospholipase C (PLC) and protein kinase C (PKC) (Figure 1A; Brignall et al., 2017). The pathways then re-converge in the nucleus, where NFAT and AP-1 bind cooperatively to activate gene expression (Chen et al., 1998, Johnson et al., 2004). Little is known about how inhibitory receptors suppress these signaling pathways and alter the T cell response. However, numerous studies point to a role for the ubiquitin ligase Cbl-b, which is activated by CTLA4 and PD-1 (Fujiwara et al., 2017, Li et al., 2004, Li et al., 2019). Cbl-b functions as a gatekeeper of T cell activation by antagonizing PLC, phosphatidylinositol 3-kinase (PI3K), and PKC signaling (Fang and Liu, 2001, Heissmeyer et al., 2004, Jeon et al., 2004). Furthermore, Cbl-b expression is raised in T cells when anergy is induced by calcineurin signaling to NFAT in the absence of PKC activation (Heissmeyer et al., 2004) or by tolerizing signals (Jeon et al., 2004).

We previously showed that a single cycle of activation of TCR/CD28 signaling pathways was sufficient to epigenetically reprogram chromatin domains encompassing immune response genes in naive T cells, resulting in the stable maintenance of altered chromatin states regulating gene accessibility and expression (Bevington et al., 2016, Bevington et al., 2017). When activated by Ag for the first time, quiescent naive T cells undergo a slow transformation into rapidly dividing T blast (T_B_) cells, during which they remodel broad chromatin domains carrying activating histone modifications and encompassing inducible genes and their regulatory elements (Figure 1B). In response to TCR signaling, these regulatory elements form open chromatin regions detectable as DNase I hypersensitive sites (DHSs). Although inducible DHSs (iDHSs) are opened up transiently by factors such as AP-1 and NFAT, and typically function as inducible transcriptional enhancers or promoters, ∼3,000 of the DHSs formed during blast cell transformation are stably maintained as primed DHSs (pDHSs) in T_B_ and in memory T cells without influencing steady-state transcription. Although these pDHSs are initially opened in response to transient activation of AP-1, they are then maintained by stable binding of constitutively expressed TFs such as ETS-1 and RUNX1, which introduce activating histone modifications into surrounding regions, thereby maintaining an epigenetic memory of TCR activation (Bevington et al., 2016, Bevington et al., 2017).

Several previous studies of anergy in the context of tolerance have investigated the roles and regulation of production of Tr1 cells, which are a Foxp3^−ve^ subset of regulatory T cells, which limit damage resulting from responses to infection and suppress auto-immunity by secreting factors such as interleukin (IL)-10 (O’Garra and Vieira, 2007, Roncarolo et al., 2014, Trinchieri, 2007). Intra-nasal or subcutaneous administration of peptides can induce an anergic IL-10-secreting Tr1-like population *in vivo* (Burton et al., 2014, Gabrysová et al., 2009, Sundstedt et al., 2003), and this treatment is effective in establishing tolerance (Burton et al., 2014) and protecting against autoimmunity (Burkhart et al., 1999, Clemente-Casares et al., 2016, Gabrysová et al., 2009, O’Neill et al., 2006). Tr1-like cells can also be generated *in vitro* by culturing T cells in IL-27 (Pot et al., 2009). The Tr1 gene expression signature resembles that of both exhausted TILs and exhausted T cells associated with chronic viral infections (Chihara et al., 2018). Tr1-like cells can also be induced by repeated anti-CD3ε antibody exposure (Mayo et al., 2016) or by nano-particles coated with peptide-bound major histocompatibility complex (MHC) class II (Clemente-Casares et al., 2016). These studies show that TCR signaling is important in generating tolerance (Wraith, 2016). However, although there is a consensus surrounding the importance of Tr1-like cells in a variety of immunological contexts, the molecular mechanisms that lead to the generation of Tr1-like tolerant cells and their altered response to Ag remain obscure.

To investigate the underlying basis of T cell tolerance, we performed genome-wide profiling of gene-regulatory networks in T cells before and after induction of tolerance, and after reactivation of TCR signaling. For this, we employed a transgenic TCR model (Tg4) (Liu et al., 1995) based on desensitization of mice in response to escalating doses of a tolerizing peptide Ag (Figure 1C; Burton et al., 2014). Tg4 mouse T cells recognize the Ac1-9 N-terminal peptide AcASQKRPSQR from myelin basic protein (MBP), an encephalitogenic auto-Ag associated with multiple sclerosis, and can be rendered tolerant by repeated exposure to the higher affinity, MHC-binding MBP Ac1-9[4Y] analog AcASQYRPSQR (4Y) (Burton et al., 2014). This approach may form the basis of future therapies in auto-immune disease because we have established that it alleviates symptoms of multiple sclerosis in patients (Chataway et al., 2018). To define epigenetic mechanisms maintaining an anergic tolerant state in Tg4 T cells, we identified DHSs on a genome-wide scale, together with genome-wide RNA-seq. These integrated studies demonstrated that the tolerized state is associated with two distinct mechanisms. First, tolerized T cells specifically maintain chromatin priming at a subset of pDHSs within archetypal T cell tolerance signature genes, allowing them to be activated at a signaling threshold below that of immune response genes. Second, receptor signaling to AP-1 is suppressed, and tolerized T cells fail to activate classic immune response genes *in vivo* in response to TCR stimulation by peptide. Hence it is altered epigenetic states that shift the balance between immune response and immune repression following tolerization.

## Results

### T Cell Tolerization Reprograms Inducible Gene Expression Potential

In the Tg4 mouse model, tolerance induction correlates with the induction of anergic CD4^+^ T cells with a Tr1 phenotype (Gabrysová et al., 2009). To address the molecular basis of this phenomenon, we first undertook genome-wide analyses of changes in gene expression (RNA sequencing [RNA-seq]) associated with tolerance in both the steady state and following challenge with a specific Ag. Tg4 transgenic mice were tolerized by repeated subcutaneous injection of the 4Y MBP peptide according to the schedule outlined in Figure 1C. To examine TCR responses in tolerized T cells (T) and in non-tolerant predominantly naive T cells (N) from control mice, we harvested cells 2 h after a final injection with 80 μg 4Y peptide (T_Ag_ and N_Ag_) or PBS vehicle (T_0_ and N_0_). We also examined *in vitro* responses of T cells after a 2-h stimulation with 20 ng/mL PMA and 2 μM calcium ionophore A23187 (T_PI_ and N_PI_) so as to distinguish between mechanisms acting at the level of TCR/CD28 signaling and transcriptional mechanisms involving inducible TFs downstream of PLC (Figure 1A). After normalization of the data, we defined 17,179 loci where the average RNA-seq value was at least 3 for at least one set of conditions (Table S1). Principle-component analysis of the 500 most variable genes using three biological replicates revealed substantial differences between naive and tolerized cells under all conditions (PC1 versus PC3; Figure 1D) and robust responses to Ag in both naive and tolerant cells (PC1 versus PC2; Figure S1A). Analysis of RNA-seq data for genes where values changed by at least 2-fold after tolerization (Figure S1B), and at least one value was above 50, revealed 475 loci higher and 107 loci lower in T_0_ than N_0_, (Table S2) and 568 loci higher and 381 loci lower in T_Ag_ than N_Ag_ (Table S3). Hierarchical clustering was performed for 637 of these genes where at least one of the T_0_:N_0_, T_Ag_:N_Ag_, T_Ag_:T_0_, or N_Ag_:N_0_ ratios showed at least a 10-fold difference (Figure S1C). The combined analyses revealed immune response genes, such as *Il2*, *Il3*, *Tnf*, *Csf2*, *Ccl1*, *Cd40lg*, *Nr4a3*, and *Nfkb1*, which were less inducible in tolerized cells treated with Ag (Figures 1E, S1C, and S1D), and anergy or tolerance-associated genes, which were more inducible, including the inhibitory receptors *Ctla4*, *Tigit*, *Lag3*, *Havcr2* (TIM3), and *Pdcd1* (PD1), the phosphatase *Dusp6*, the TFs *Nfil3* and *Maf*, and the immune-suppressive cytokines *Il10* and *Il21* (Figures 1F, S1C, and S1E). For many immune response genes, this loss of responsiveness to TCR signaling could be bypassed by direct stimulation with PI (Figures 1E and S1D), suggesting that tolerance involves a membrane-proximal block in TCR signaling to inducible TFs. In contrast, the weak response to TCR signaling seen for many immuno-suppressive genes in naive cells could not be boosted by PI. Similar responses were seen over an extended time course of stimulation, demonstrating that it is not just the kinetics but the magnitude of activation that varies (Figures S1F and S1G). Some genes were also expressed at higher levels in tolerized cells even prior to stimulation (Figures 1F, S1C, and S1E; Table S2). Many of the genes upregulated in T_0_ had also been previously shown to be upregulated in Tr1-like cells generated *in vitro* with IL-27, suggesting similarities between these two model systems (Chihara et al., 2018; Table S4). These include *Maf*, *Il10*, *Il21*, *Havcr2*, *Lag3*, *Nfil3*, and *Prdm1*. These data suggest a potential model whereby tolerized T cells exhibit both: (1) suppression of TCR/CD28 signaling to a level below the threshold needed for activation of many immune response genes; and (2) a heightened sensitivity of immuno-regulatory genes that still allows activation at reduced levels of TCR signaling, consistent with the actions of inhibitory receptors such as PD-1 (Shimizu et al., 2020). Significantly, TCR-inducible activation of *Il2*, *Csf2*, and *Tnf* is dependent on CD28 signaling (Yukawa et al., 2020).

### Modification of the Epigenetic Landscape in Tolerant Cells

**Figure 2:**
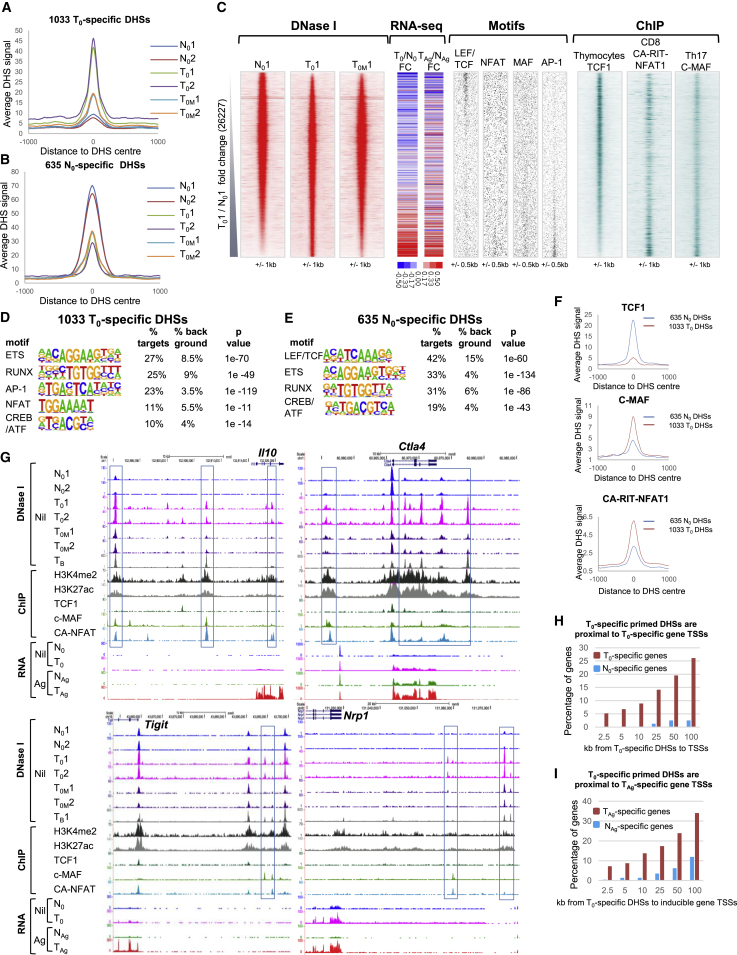
DNase-Seq Analyses of Open Chromatin in Naive and Tolerized T Cells (A and B) Average DHS signal at the 1,033 T_0_-specific DHSs (A) and at the 635 N_0_-enriched DHSs (B) in the replicate N_0_, T_0_, and T_0M_ samples. (C) DNase-seq sequence tag density plots showing all 26,227 peaks detected in replicate 1 of T_0_ and N_0_ ordered by increasing fold change (FC) of sequence tag count for T_0_ compared with N_0_. Alongside is the FC in mRNA level for the closest gene to the DHSs as ranked in the density plots. The FC is shown between T_0_ and N_0_ (left), and T_Ag_ is compared with N_Ag_ (right). The locations of TF consensus binding motifs within the same DHSs are shown alongside for AP-1, NFAT, MAF, and LEF family proteins. Plotted alongside are published TF binding data from ChIP-seq analyses for TCF1 in thymocytes (Dose et al., 2014), c-MAF in Th17 cells (Ciofani et al., 2012), and ectopic CA-RIT-NFAT1 in CD8 T cells (Martinez et al., 2015). (D and E) HOMER *de novo* identification of TF motifs enriched in the 1,033 T_0_-specific DHSs (D) and the 635 N_0_-specific DHSs (E). (F) Average ChIP signal at the 1,033 T_0_-specific DHSs and 635 N_0_-specific DHSs for TCF1, c-MAF, and CA-RIT-NFAT1. (G) UCSC Genome Browser tracks showing DNase-seq data from replicate samples of non-stimulated naive cells (N_0_), non-stimulated tolerant cells (T_0_), and tolerant cells 3 weeks after the final dose of peptide (T_0M_), plus published DNase-seq data for CD4 T blast (T_B_) cells (Bevington et al., 2016). Published ChIP-seq data are shown for the TFs TCF1, c-MAF, and CA-RIT-NFAT1 and the histone modifications H3K27ac and H3K4me2 in T_B_ cells. Representative RNA-seq data from one of three replicates are also shown for resting and *in vivo* peptide-stimulated naive and tolerant cells (0 and Ag). (H and I) Bar graphs showing the percentage and proximity of genes that are preferentially induced in T_0_ or N_0_ (H) and T_Ag_ or N_Ag_ (I), which are found within 100 kb of the 1,033 T_0_-specific DHSs.

We next investigated whether the more efficient activation of a subset of genes in tolerant cells was due to epigenetic reprogramming at the level of chromatin modifications, as observed in T_B_ cells (Bevington et al., 2016). To this end, we globally identified DHSs in N_0_ and T_0_ cells using DNase sequencing (DNase-seq). We ranked the DHSs based on the fold change of DNase-seq signal between N_0_ and T_0_ for two independent replicates (Figure S2A). We selected high-confidence subsets of DHSs that were at least 2-fold different in both experiments. These analyses identified 1,033 T_0_-specific DHSs (tDHSs) and 635 N_0_-specific DHSs (Figures 2A, 2B, and S2A; Data S1). We also used DESeq2 as an alternative method of statistical analysis to screen for 2-fold changes and p < 0.05, and again identified the vast majority of the 1,033 tDHSs defined above (84%; Figure S2B).

We also investigated the stability of the tDHSs after a time interval sufficient for peak effector T cell responses to have subsided and for memory T cells to form (T_0M_, Figure 1C). We performed DNase-seq on tolerized cells that had received the final dose of Ag 3 weeks prior to harvest, and we still reproducibly detected over half of the 1,033 tDHSs (Figure S2C) that maintained an elevated average DHS signal (Figures 2A and 2C). These data suggest that tolerization involves long-term maintenance of epigenetic reprograming of a specific subset of DHSs in the absence of sustained TCR signaling. To ascertain whether the changes in chromatin structure had any impact on the nearby genes, we measured the difference in mRNA expression for the genes closest to the ranked DHSs for replicate 1 (Figure 2C). We observed an increase in both steady-state (T_0_ compared with N_0_) and inducible (T_Ag_ compared with N_Ag_) gene expression in parallel with the increase in DHS strength. This is consistent with a role for epigenetic priming in the imprinting of a transcriptional memory of TCR activation, thereby enabling both higher steady-state expression in T_0_ and more efficient re-activation in T_Ag_ compared with N_Ag_.

To gain insight into transcriptional mechanisms underlying tolerization, we used HOMER to perform *de novo* identification of TF motifs in the different specific subsets of DHSs (Figures 2D, 2E, and S2B). The tDHSs were more highly enriched in AP-1 and NFAT motifs, whereas the pre-existing N_0_-specific DHSs contained a higher proportion of LEF/TCF motifs. Both subsets were enriched in RUNX, ETS, and CREB/ATF motifs. The distribution of AP-1, NFAT, MAF, and LEF/TCF motifs is depicted in Figure 2C, where their direct correlation with the coordinates of the DHSs is shown. Published chromatin immunoprecipitation sequencing (ChIP-seq) data of TCF1 binding in thymocytes was consistent with these *in silico* observations (Dose et al., 2014), with TCF1 binding concentrated in both the N_0_-specific and shared peaks but depleted in the tDHSs (Figures 2C and 2F). Conversely, MAF binding was more enriched in the tDHSs than the N_0_-specific DHSs (Ciofani et al., 2012; Figures 2C and 2F). MAF has been shown to be important in regulating *Il10* expression (Xu et al., 2009) and is implicated in repressing *Il2* (Gabryšová et al., 2018). Maf is also upregulated in T_0_ and T_Ag_ cells (Figure 1F), and MAF can dimerize with other CREB/AP-1 family proteins to bind to AP-1 sites (Kataoka et al., 1994), a motif that is enriched in the tDHSs (Figures 2C and 2D). The enrichment of NFAT motifs in tDHSs may also be functionally relevant because NFAT plays a role in T cell anergy (Macián et al., 2002) and T cell exhaustion (Martinez et al., 2015). Indeed, when we aligned our DNase-seq data with ChIP-seq data generated using a constitutively active modified form of NFAT1/NFATC2 (CA-RIT-NFAT), which is known to promote exhaustion (Martinez et al., 2015), we observed that NFAT binding was more strongly enriched in the tDHSs (Figures 2C and 2F). Although it is unlikely/unknown whether NFAT is binding to these sites in T_0_ cells before stimulation, the presence of the motif at an open DHS should enable rapid binding when the cells are re-exposed to Ag.

Examples of tDHSs with binding sites for MAF and NFAT were found at the tolerance-associated *Il10*, *Ctla4*, *Tigit*, and *Nrp1* loci (Figure 2G), where there are higher levels of either constitutive or inducible mRNA expression in tolerized cells (Figures 1F and 2G). At these loci, tDHSs were present in T_0_, but not in N_0_, and these sites were largely still maintained 3 weeks later in T_0M_. The tDHSs were more likely to bind CA-RIT-NFAT and/or c-MAF, but not TCF1. Furthermore, recently activated CD4 T_B_ cells generated by *in vitro* activation exhibit many of the same DHSs as tolerant cells, and these regions are flanked by the active chromatin modifications H3K27ac and H3K4me2 in T_B_ cells (Figure 2G). It is likely that the tDHSs in tolerant cells will be similarly embedded within extensive active chromatin domains that have been formed during the tolerization process.

### T_0_-Specific DHSs Are Proximal to T_0_- and T_Ag_-Specific Genes

Previously activated T cells maintain transcriptional memory by employing pDHSs to establish active chromatin domains encompassing adjacent inducible regulatory elements (Figure 1B). To assess the roles of the equivalent tDHSs in the regulation of inducible gene expression in tolerant cells, we integrated the mRNA data for differentially expressed genes with the differential DHS peak data. Starting with the gene sets for mRNA values greater than 50 defined in Figure S1B, we selected the annotated genes that have been assigned to a specific genomic locus, and further selected inducible genes on the basis that the T_Ag_- and N_Ag_-specific genes are also 2-fold inducible compared with T_0_ and N_0_, respectively. This allowed us to define genes expressed higher in T_0_ compared with N_0_ (460), N_0_ compared with T_0_ (80), and inducible genes expressed higher in T_Ag_ compared with N_Ag_ (138) and in N_Ag_ compared with T_Ag_ (226) (Table S5). We then measured the distances from the transcription start sites (TSSs) of differentially expressed genes to the closest of the 1,033 tDHSs (Figures 2H and 2I) and the closest of the 635 N_0_-specific DHSs (Figures S2D and S2E). These analyses revealed that the 1,033 tDHSs were preferentially located closer to the TSSs of T_0_- and T_Ag_-specific genes, with approximately 25% of the T_Ag_-specific genes located within 50 kb of a tDHS (Figure 2I). Hence these data suggest that tDHSs do indeed prime closely linked inducible loci for rapid re-activation in cells re-exposed to Ag. For example, tDHSs exist at the *Il10*, *Ctla4*, and *Tigit* genes, which are more strongly induced in T_Ag_ than in N_Ag_ (Figure 2G).

In contrast, priming seems to play little role in defining inducible responses in naive cells, with only 10% of the 226 N_Ag_-specific gene TSSs within 50 kb of N_0_-specific DHSs (Figure S2D). Instead, the loss of DHSs in N_0_ correlates more closely with a loss of expression in N_0_, whereby 25% of the 80 N_0_-specific genes existed within 50 kb of N_0_-specific DHSs (Figure S2E). An example of this is shown at the naive T cell-specific gene *Satb1* (Figure S2F), which has been implicated in repressing *Pdcd1* (PD-1) expression in T cells (Stephen et al., 2017). Notably, the DHSs at *Satb1* can bind TCF1, consistent with the enrichment of LEF/TCF motifs at the 635 N_0_-specific sites.

### Activation of Signaling Pathways Induces a Different DHS Profile in Tolerant Cells

**Figure 3:**
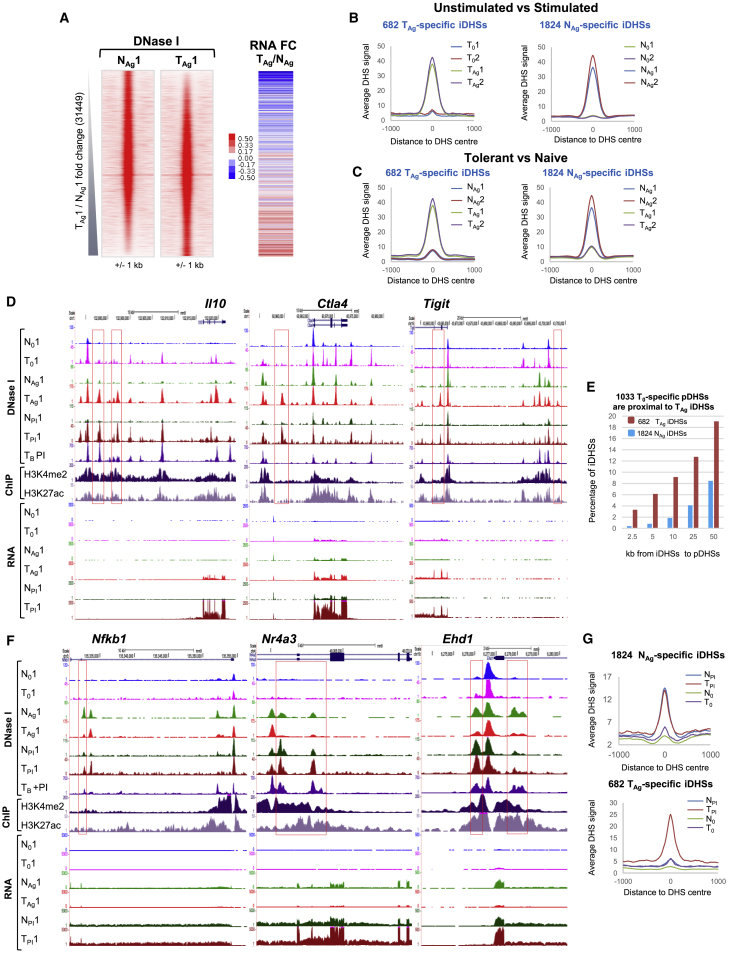
DNase-Seq Analyses of *In Vivo*-Activated Naive and Tolerized T Cells (A) DNase-seq tag density plots showing all peaks detected in replicate 1 of N_Ag_ and T_Ag_ ranked according to FC of sequence tag count for T_Ag_ compared with N_Ag_. Alongside is the FC in mRNA level for the closest gene to the DHSs for T_Ag_ compared with N_Ag_. (B and C) Average DHS signal at the 682 T_Ag_-specific iDHSs (left) and at the 1,824 N_Ag_-specific iDHSs (right) for 0 compared with Ag (B) and N_Ag_ compared with T_Ag_ (C). (D) UCSC genome browser tracks showing DNase-seq and RNA-seq data from resting cells (N_0_ and T_0_), antigen-stimulated cells (N_Ag_ and T_Ag_), and PI-stimulated cells (N_PI_ and T_PI_). DNase-seq, H3K27ac ChIP, and H3K4me2 ChIP tracks are shown for CD4 T_B_ PI. (E) Bar graph showing the percentage of iDHSs that are preferentially induced in T_Ag_ or N_Ag_ that are found within 50 kb of the 1,033 tDHSs. (F) UCSC genome browser tracks as for (D). (G) Average DNase-seq signal for the 1,824 N_Ag_-specific iDHSs and the 682 T_Ag_-specific iDHSs in non-stimulated (N_0_ and T_0_) and PI-treated (N_PI_ and T_PI_) cells.

To investigate whether epigenetic priming at tDHSs supports inducible chromatin remodeling in tolerant T cells, we performed DNase-seq on N_Ag_ and T_Ag_, and identified the differential DHSs by ranking the regions based on the fold change of signal (Figures 3A and S3A). We confirmed that the formation of T_Ag_-specific DHSs was generally correlated with increased gene expression by measuring the difference in mRNA expression in T_Ag_ compared with N_Ag_ for the genes that are adjacent to the corresponding DHSs (Figure 3A). To define high-confidence subsets of specific DHSs, we intersected the peaks that were at least 2-fold enriched in each sample for two independent pairs of replicates. This gave 2,680 N_Ag_-specific peaks and 1,959 T_Ag_-specific peaks (Figure S3A). As we were specifically interested in iDHSs, we filtered them further to include only peaks that were at least 3-fold higher in N_Ag_ compared with N_0_, or in T_Ag_ compared with T_0_ (Figures S3B and S3C). This revealed 1,824 N_Ag_-specific iDHSs and 682 T_Ag_-specific iDHSs (Data S1; Figure 3B), which were highly specific for just naive or tolerant cells (Figure 3C). The same sets of specific DHSs were also defined by a parallel analysis using DESeq2 and the same fold change for each subset (p < 0.05), which identified 2,161 N_Ag_-specific iDHSs and 923 T_Ag_-specific iDHSs, including 88% of the 1,824 N_Ag_ sites and 90% of the 682 sites T_Ag_ (Figure S3D). Examples of T_Ag_-specific iDHSs can be observed at the tolerance-associated *Il10*, *Tigit*, and *Ctla4* loci (Figure 3D), where iDHS induction correlated with increased mRNA gene expression in T_Ag_ cells. Furthermore, 19% of the T_Ag_-specific iDHSs were located within 50 kb of a tDHS (Figure 3E), consistent with locus priming enabling more efficient activation of closely linked enhancers residing in the same active chromatin domain (Figure 1B). Many of the same iDHSs that are induced in T_Ag_ are also induced in T_B_ cells stimulated with PI (Figure 3D). In Figures 2G and 3D, it is also evident that regions associated with iDHSs induced by PI in T_B_ cells were already marked with H3K4me2 and H3K27ac modifications before Ag stimulation. In contrast with tolerant cells, naive T cells are not preferentially primed by pDHSs at the N_Ag_-specific genes in N_0_ cells, but nevertheless still induce N_Ag_-specific iDHSs (Figure 3F).

### N_Ag_-Specific iDHSs Can Be Induced in Tolerant Cells by Bypassing TCR/CD28 Signaling

The treatment of tolerant cells with PI enabled the induction of many N_Ag_-specific genes in tolerant cells, whereas the T_Ag_-specific genes could not be induced by PI in naive cells (Figures 1E and 1F). To determine whether this same trend was observed at the chromatin level, we measured DNase I accessibility in naive and tolerant cells treated with PI. The mRNA induction correlated closely with the changes in chromatin structure whereby T_Ag_-specific iDHSs could not be induced in naive cells, most likely due to the lack of epigenetic priming, whereas N_Ag_-specific iDHSs were induced in tolerant cells treated with PI. This was observed at both the local level (Figures 3D and 3F) and globally at the 1,824 N_Ag_ and 682 T_Ag_ iDHSs (Figure 3G). The ability of PI to overcome the block in tolerant cells is consistent with suppression of TCR signaling upstream of PLC and PKC to inducible TFs, a block that could be bypassed by direct activation of PKC and RAS signaling by PI (Figure 1A).

### N_Ag_- and T_Ag_-Specific iDHSs Are Governed by Distinct Gene-Regulatory Networks

**Figure 4:**
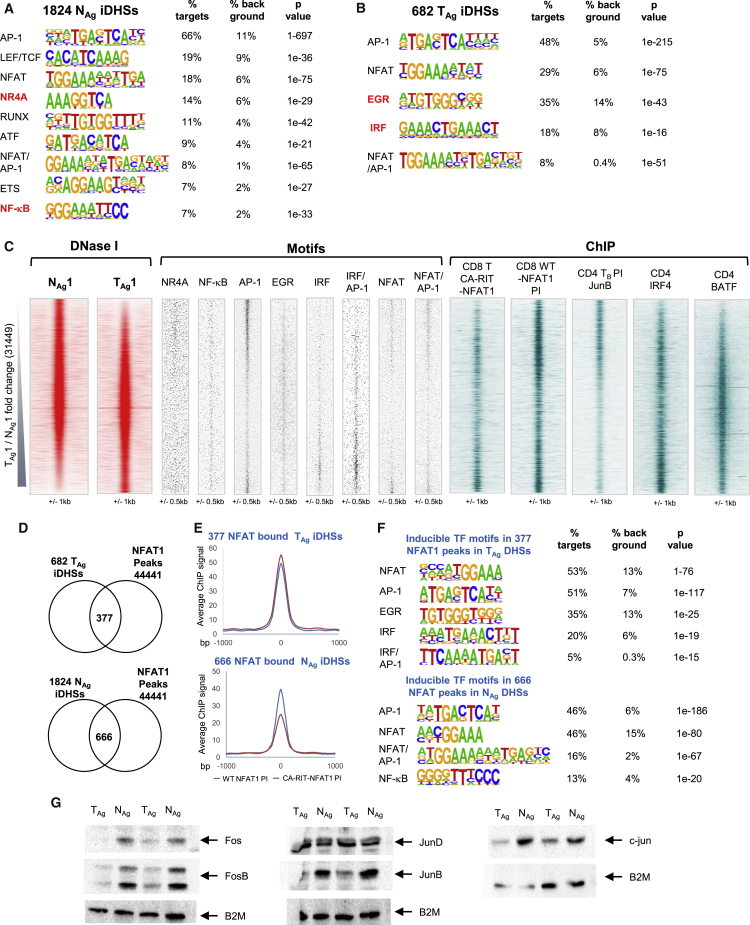
Analyses of TF Interactions with DHSs in Naive and Tolerized T Cells (A and B) Homer *de novo* identification of enriched TF motifs in the 1,824 N_Ag_-specific iDHSs (A) and the 682 T_Ag_-specific iDHSs (B). (C) Locations of transcription factor binding motifs (middle) at all DNase I peaks in the Ag-stimulated samples ordered according to the FC in sequence tag density for T_Ag_ compared with N_Ag_ (left). Aligned on the same axis are published ChIP-seq data for JunB in T_B_ cells (Bevington et al., 2016), IRF4, BATF in CD4 T cells (Li et al., 2012) and constitutively active CA-RIT-NFAT and endogenous PI-induced NFAT in CD8 T cells (Martinez et al., 2015) (right). (D) Venn diagrams depicting the overlaps between the published data for 4,4441 peaks detected in ChIP assays for CA-RIT-NFAT1 and/or endogenous (WT) NFAT1 in PI-stimulated T cells (Martinez et al., 2015) with the 682 T_Ag_-specific iDHSs (upper) or 1,824 N_Ag_-specific iDHSs (lower). (E) Average ChIP-seq signal for WT NFAT1 and CA-RIT-NFAT in PI-stimulated T cells at the 377 NFAT T_Ag_-specific iDHSs (upper) and 666 NFAT-bound N_Ag_-specific iDHSs (lower). (F) HOMER *de novo* identification of inducible TF motifs within the 377 and 666 sites. (G) Representative example of western blot assays of Fos, FosB, JunB, Jun, JunD, and B2M proteins in T cells.

Tolerance involves the expression and activation of inhibitory receptors that antagonize TCR/CD28 signaling to the inducible TFs, which create iDHSs. To identify gene-regulatory signatures within the specific subsets of N_Ag_ and T_Ag_ DHSs, we performed HOMER *de novo* motif analyses of enriched TF motifs (Figures 4A and 4B) and plotted the positions of the identified motifs over the DHS coordinates, together with published ChIP-seq data for these same TFs (Figure 4C). The N_Ag_-specific iDHSs had a complex TF motif signature reflecting the TCR/CD28-inducible TFs AP-1, NFAT, NF-κB, and NR4A, but not EGR or IRF family TFs (Figures 4A and 4C). The T_Ag_-specific DHSs were enriched for EGR and IRF motifs, but not motifs for the TCR/CD28-induced TFs NF-κB and NR4A. The latter could be explained in part by reduced expression for *Nr4a3* and *Nfkb1* in T_Ag_ compared with N_Ag_ (Figure 1E) and the absence of N_Ag_-specific iDHSs induced at these loci (Figure 3F). These analyses were also confirmed using DESeq2, which identified essentially the same subsets of N_Ag_ and T_Ag_ DHSs with the same motif composition as those identified by the above pairwise analysis (Figures S4A and S4B).

### Evidence for Distinct AP-1, NFAT, and IRF Complexes in T_Ag_ and N_Ag_ Cells

Although the T_Ag_ and N_Ag_ iDHSs both contained NFAT and AP-1 motifs, the T_Ag_-specific iDHSs were more enriched for NFAT motifs, whereas the N_Ag_ iDHSs had a higher proportion of AP-1 binding motifs (Figures 4A–4C). Consistent with this, published ChIP-seq data reveal higher levels of binding of the constitutively active CA-RIT-NFAT (Martinez et al., 2015) at the T_Ag_-specific iDHSs and higher levels of JunB AP-1 binding at the N_Ag_-specific iDHSs in T_B_ cells stimulated with PI (Bevington et al., 2016; Figure 4C). Because CA-RIT-NFAT has modifications that prevent cooperative interactions with AP-1, these data may indicate that much of the NFAT binding seen at T_Ag_-specific DHSs is independent of AP-1, as seen in other models of exhaustion and in TILs (Macián et al., 2002, Martinez et al., 2015, Mognol et al., 2017). In contrast, published ChIP-seq data for endogenous NFAT1 (NFATc2) in CD8 T cells stimulated with PI, where AP-1 will be efficiently induced (Martinez et al., 2015), revealed NFAT binding to many N_Ag_-specific iDHSs that are unable to bind CA-RIT-NFAT (Figure 4C), suggesting direct cooperation between NFAT and AP-1.

To investigate NFAT further, we identified all 44,441 ChIP peaks that could bind either CA-RIT-NFAT1 or wild-type (WT) NFAT1, and then intersected these with the 1,824 N_Ag_ and 682 T_Ag_ iDHSs (Figure 4D). This analysis revealed that 55% of the 682 T_Ag_ iDHSs (377 sites) were capable of binding at least one form of NFAT, whereas just 36% of the N_Ag_-specific iDHSs could bind NFAT. The average level of binding of CA-RIT-NFAT1 detected at the N_Ag_ iDHSs was also weaker than endogenous NFAT1 binding (Figure 4E), again suggesting that cooperative binding with AP-1 is more important at N_Ag_ iDHSs. This concept was further supported by an enrichment of the composite NFAT/AP-1 element detected by HOMER in the N_Ag_, but not the T_Ag_, NFAT binding sites (Figure 4F). Examples of these patterns of binding were observed at the T_Ag_-specific genes *Tigit* and C*tla4*, where the T_Ag_-specific iDHSs bind NFAT independently of AP-1 (CA-RIT NFAT) (Figure S4C). In contrast, the N_Ag_-specific genes *Fosl2* and *Nfkb1* bind WT NFAT only when the cells are stimulated with PI and AP-1 is present (WT NFAT PI) (Figure S4D). Furthermore, consensus motifs for cooperative NFAT/AP-1 binding were found at the N_Ag_-specific genes, whereas the T_Ag_-specific iDHSs lacked the true composite element with correctly spaced NFAT and AP-1 motifs (Chen et al., 1998). The above observations and conclusions were supported by a reduction in the total amount of the activating AP-1 proteins c-Fos, Fosb, c-Jun, and JunB in T_Ag_ compared with N_Ag_, whereas levels of JunD were not reduced (Figure 4G). JunD has immuno-suppressive functions in lymphocytes (Meixner et al., 2004), suggesting that the ratio between JunD and other activating AP-1 proteins may influence the activity of AP-1 target genes in tolerized cells.

JunD has previously been detected in AP-1/IRF complexes in T cells (Li et al., 2012). This is significant here because IRF family proteins also play a role in anergy, in association with BATF/Jun AP-1-like complexes, whereby these factors can bind to composite IRF1/AP-1 motifs, including at the *Il10* locus (Glasmacher et al., 2012, Li et al., 2012). Although the composite IRF/AP-1 motif was not initially detected in the 682 T_Ag_ iDHSs (Figure 4B), a direct search detected it in 12% of these sites, compared with less than 3% of the 1,824 N_Ag_ iDHSs, and the IRF/AP-1 motif was identified by HOMER within the specific subset of 377 NFAT-bound N_Ag_ iDHSs (Figure 4F). Furthermore, BATF has been identified in IRF/AP-1 complexes in anergic Tr1 cells (Karwacz et al., 2017), and here we observed that published ChIP peaks for both BATF and IRF4 are enriched in T_Ag_, but not N_Ag_, DHSs (Figure 4C).

### TF Occupancy Differs in Tolerant and Naive Cells

**Figure 5:**
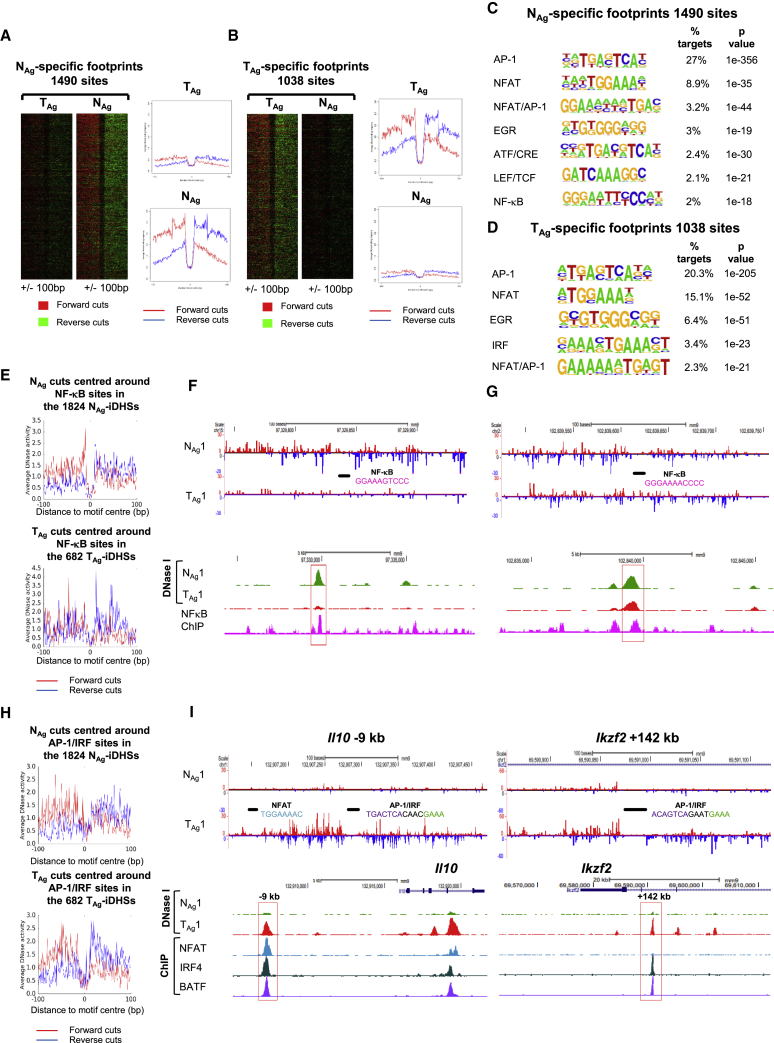
Footprinting Analyses of TF Occupation in Naive and Tolerized T Cells (A and B) Footprints within (A) N_Ag_- and (B) T_Ag_-specific iDHSs ordered according to the Wellington footprinting occupancy score. (C and D) HOMER *de novo* identification of TF motifs enriched within footprints in the 1,824 N_Ag_-specific iDHSs (C) and the 682 T_Ag_-specific iDHSs (D). (E) Average profile showing the DNase I cuts around all NF-κB sites in the 1,824 N_Ag_-specific iDHSs in N_Ag_ (upper) and 682 T_Ag_-specific iDHSs in T_Ag_ (lower). (F and G) Examples of Wellington digital footprinting of DNase-seq data showing protection of an NF-κB site at a N_Ag_-specific iDHS (F) and at a shared iDHS (G). DNase-seq data are shown for N_Ag_ and T_Ag_, and ChIP-seq data for NF-κB (Oh et al., 2017). (H) Average profile showing the DNase I cuts around all AP-1/IRF sites in the 1,824 N_Ag_-specific iDHSs in N_Ag_ (upper) and 682 T_Ag_-specific iDHSs in T_Ag_ (lower). (I) Examples of Wellington digital footprinting of DNase-seq data showing protection of NFAT and composite AP-1/IRF motifs. DNase-seq data are shown for N_Ag_ and T_Ag_, and ChIP-seq data for NFAT1 (Martinez et al., 2015), IRF4, and BATF (Li et al., 2012).

To look for further evidence of differential TF occupancy, we generated higher read depth DNase-seq data for one sample from each group and performed digital DNase I footprinting of protected TF motifs. Using the Wellington footprinting algorithm (Piper et al., 2013), we identified 1,490 protected sites in N_Ag_-specific iDHSs and 1,038 protected sites in T_Ag_-specific iDHSs (Figures 5A and 5B). Consistent with the results depicted in Figure 4, more AP-1 sites were occupied in N_Ag_ compared with T_Ag_, and more NFAT and EGR sites were protected in T_Ag_ compared with N_Ag_, while NF-κB and IRF sites were protected only in N_Ag_ and T_Ag_, respectively (Figures 5C and 5D). To further validate these results, we plotted the forward and reverse DNase I cuts surrounding all the NF-κB motifs that were present in the 1,824 N_Ag_-iDHSs and 682 T_Ag_-iDHSs (Figure 5E). NF-κB sites in the 1,824 iDHSs were more protected in the N_Ag_ sample, indicating that this TF is most likely bound here. Examples are shown in Figures 5F and S5A, where publically available ChIP-seq data (Oh et al., 2017) show NF-κB binding correlating with these footprints. Furthermore, in some cases where an NF-κB motif was present within an iDHS that was induced in both N_Ag_ and T_Ag_, more efficient protection was observed in the N_Ag_ cells (Figures 5G and S5B). This further suggests a reduction in signaling to NF-κB in tolerized cells.

We similarly investigated the occupancy of AP-1/IRF motifs and determined that these composite motifs had a greater level of protection in the 682 T_Ag_-specific iDHSs than in the 1,824 N_Ag_ iDHSs (Figure 5H). The T_Ag_-specific genes *Il10*, *Ikzf2*, and *Nrp1* loci each encompass strongly protected AP-1/IRF motifs (Figures 5I and S5C). Furthermore, ChIP-seq data from previously published studies (Li et al., 2012) showed that these sites could bind both IRF4 and BATF (Figures 5I and S5C). At the *Il10 −*9 kb and *Nrp1* +177 kb DHSs, in addition to IRF4 and BATF binding, NFAT peaks were identified in published ChIP data for CA-RIT-NFAT, at sites where NFAT motifs were footprinted in T_Ag_ cells, reinforcing the role of these three factors in gene-regulatory networks in tolerized T cells as has been shown in exhausted T cells (Man et al., 2017).

### TCR Signaling Complexes Are Disrupted in Tolerized T Cells

**Figure 6:**
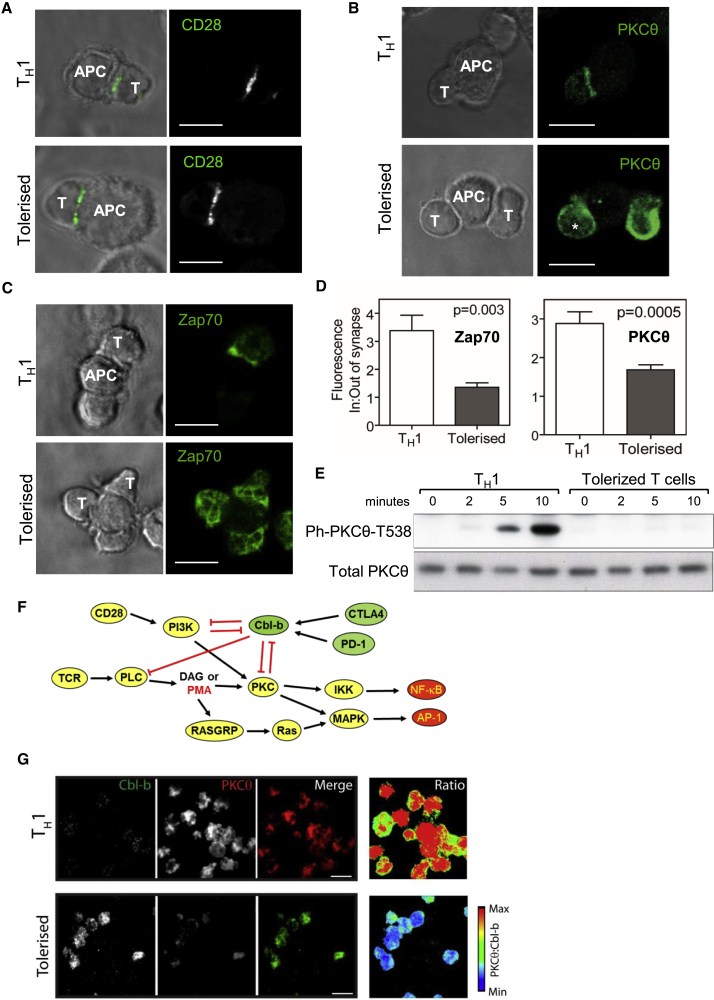
Altered Immunological Synapse Morphology and Reduced TCR-Proximal Signaling in Tolerant T Cells T cells were tolerized by either intra-nasal (IN) delivery of peptides (A–E and G) or sub-cutaneous injection of peptides (F). (A–C) Confocal microscopy of Th1-like cells or IN-tolerized T cells coupled with bone marrow APCs presenting cognate peptide for 10 min and imaged by immunofluorescence confocal microscopy following immune-labeling for (A) CD28, (B) PKCθ, or (C) Zap70. (D) Quantification of confocal microscopy images showing mean enrichment of PKCθ and Zap70 at the T cell-APC interface as in (B) and (C). Values were derived from at least three biological replicates. p values were calculated by Mann-Whitney test, error bars are SEM. (E) Western blot analyses of PKCθ T538 phosphorylation in Tg4 Th1 or IN-tolerant T cells following activation with cross-linked anti-CD3/CD28 for the indicated amounts of time. (F) Model depicting TCR and CD28 signaling pathways that can be antagonized when Cbl-b is activated by CTLA4 or PD-1, or bypassed by PMA. (G) TIRF microscopy of Cbl-b and PKCθ enrichment at the T cell immunological synapse in Th1-like cells or tolerized T cells. Scale bars: 10 μM.

To look for direct evidence of defective signaling in tolerant cells, we used microscopy to investigate TCR signaling molecules at the immune synapse formed after engagement of antigen-presenting cells (APCs) with either *in vitro*-generated T helper 1 (Th1) cells or T cells tolerized by repeated intra-nasal delivery of the 4Y peptide (Figure 6). We investigated the localization of Zap70 and PKCθ just downstream of TCR and PKC signaling (Figure 1A). These analyses revealed that although CD28 clustering at the TCR synapse occurs in both Th1 and tolerized T cells, Zap70 and PKCθ can no longer be efficiently recruited to the synapse in tolerized T cells (Figures 6A–6C). Quantitation of the proportion of signal at the synapse confirmed that there was a global decrease in the amount of Zap70 and PKCθ that migrates to the synapse upon engaging APCs in tolerized T cells compared with Th1 cells (Figure 6D). This breakdown of signaling from the synapse correlated with a reduction of PKCθ T538 phosphorylation in tolerized cells (Figures 6E). We also assessed the opposing roles played by PKCθ and Cbl-b, which functions as the gatekeeper of TCR/CD28 signaling (Li et al., 2004, Qiao et al., 2013, Zhang et al., 2002; Figure 6F). Total internal reflection fluorescence (TIRF) microscopy revealed lower levels of Zap70 and PKCθ in the same plane as the TCR in tolerized T cells engaged with surface-bound CD3 Abs and higher levels of Cbl-b, compared with Th1 cells (Figures S6A–S6D). There was a negative correlation between PKCθ and Cbl-b co-localizing in activated T cells for Th1 and tolerized T cells (Figure 6G). Significantly, PKCθ is just downstream of signaling from CD28 and PI3K, and both PI3K and PKC are repressed by Cbl-b (Figure 6F). PKCθ is also a direct target of PMA, and Ras proteins are indirect targets of PMA, thereby accounting for the ability of PMA to overcome the block in signaling imposed by CTLA4, PD-1, and Cbl-b (Figure 6F). Furthermore, a previous study found that ubiquitination of the p85 subunit of PI3K by Cbl-b blocks co-association of CD28 and the TCR, and thereby suppresses TCR signaling (Fang and Liu, 2001).

## Discussion

### A Two-Step Model Accounting for T Cell Tolerance

**Figure 7:**
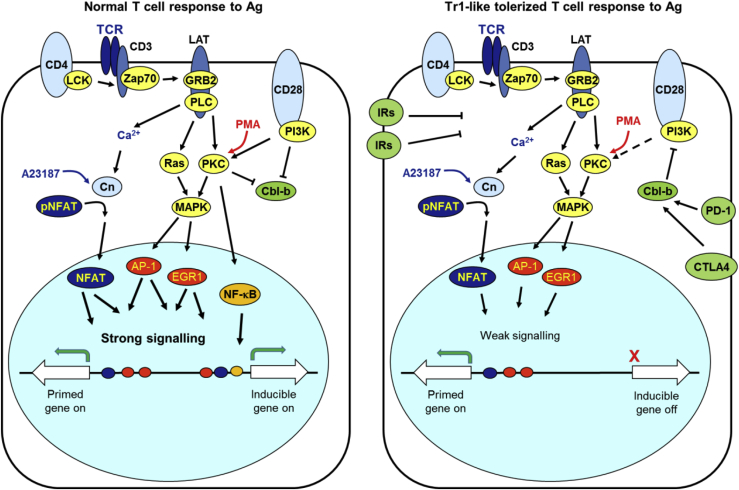
A Two-Step Model Accounting for T Cell Tolerance In a normal T cell immune response, efficient co-activation of TCR and CD28 signaling leads to strong activation of inducible TFs and immune response genes. In tolerized T cells, inhibitory receptors activate repressive factors, such as the ubiquitin ligase Cbl-b, which weaken but do not eliminate TCR/CD28 signaling. This reduced level of signaling is sufficient to epigenetically prime and activate inhibitory receptor genes, but not most immune response genes.

Increasing evidence shows that overactive immune responses in allergy and autoimmunity can be corrected by Ag-specific immunotherapy. We established that cells exposed to soluble peptide epitopes become anergic and switch from a pro-inflammatory phenotype to an anti-inflammatory Tr1-like phenotype (Burkhart et al., 1999, Sundstedt et al., 2003). These Foxp3^−ve^ T cells are anergic and capable of suppressing other immune cells through secretion of IL-10, which inhibits the Ag-presenting machinery of APCs such as dendritic cells (Gabrysová et al., 2009). In the current study, our genomic analyses utilized an *in vivo* model to answer many of the outstanding questions about the epigenetic mechanisms underlying and maintaining tolerance. Building upon published data, we propose a comprehensive inter-connected model explaining T cell tolerance: (1) the increased activity of inducible inhibitory receptors lowers TCR/CD28 signaling strength below the threshold required for induction of many immune response genes; and (2) epigenetic reprograming of inhibitory receptor genes allows them to be induced at a lower threshold of TCR/CD28 signaling than that required for immune response genes (Figure 7). Therefore, immune response genes, such as *Il2*, *Csf2*, *Tnf*, *Cd40lg*, *Nr4a3*, and *Ccl1*, can be rapidly induced in naive T cells and in effector T cells, but not in tolerized T cells. The activation of TCR signaling in the absence of CD28 co-stimulation also leads to loss of activation of *Il2*, *Csf2*, and *Tnf* (Yukawa et al., 2020), consistent with suppression of CD28 signaling in tolerized cells. Some of the immune response genes induced in naive T cells, such as *Nr4a3* (Figure 3F), are not epigenetically primed, and thus require an elevated threshold of signaling for their induction. Conversely, epigenetic priming creates an accessible chromatin environment at immuno-suppressive genes such as *Ctla4* and *Il10*, meaning that they can still be induced in tolerized cells in the presence of reduced levels of TCR/CD28 signaling (Figure 7). However, these genes are not yet primed in naive T cells, meaning that their original epigenetic state was altered after the first round of activation by epigenetic priming at DHSs in close proximity to inducible enhancers and promoters, allowing more efficient induction as described by us previously for memory T cells and T_B_ cells (Bevington et al., 2016).

### Inhibitory Receptors Suppress TCR/CD28 Signaling in Tolerized Cells

We demonstrated that tolerization leads to both high steady-state levels and higher inducible levels of inhibitory receptors. The nature of the suppressive pathways downstream of most inhibitory receptors, including TIM3, LAG3, and TIGIT, still remains poorly understood. However, it is known that both CTLA4 (Li et al., 2004, Li et al., 2019) and PD-1 (Fujiwara et al., 2017) mediate activation of the repressive ubiquitin ligase Cbl-b, and our data suggest a role for Cbl-b in limiting genomic responses in the tolerant state. Cbl-b functions in setting the threshold of T cell activation and is essential for both anergy (Tang et al., 2019, Zhang et al., 2002) and the development of inducible regulatory T cells (Qiao et al., 2013). In its absence, the response to TCR signaling is uncoupled from co-receptor signaling, and mice develop spontaneous auto-immunity (Bachmaier et al., 2000). In the anergic state associated with tolerance and exhaustion, Cbl-b represses TCR/CD28 signaling by: (1) targeting the p85 subunit of PI3K for ubiquitination and blocking its association with CD28 (Fang and Liu, 2001); (2) directing ubiquitination of PKCθ and targeting it for degradation (Heissmeyer et al., 2004); and (3) directing the ubiquitination of PLCγ1, blocking its activation (Jeon et al., 2004; Figure 7). Conversely, CD28 signaling promotes ubiquitination and degradation of Cbl-b in effector T cells (Zhang et al., 2002). Furthermore, effector T cells normally express Satb1, which represses *Pdcd1* (PD-1) expression (Stephen et al., 2017). Our data show that *Pdcd1* activation in tolerized T cells occurs in parallel with repression of *Satb1*, and we confirmed that suppressive pathways involving Cbl-b have been triggered in tolerized cells. Others have also recently demonstrated that PD-1 engagement in T cells leads to loss of expression of genes that rely on a high threshold of signaling, such as *Il2, Il3*, *Csf2*, *Ccl1*, and *Cd40lg* (Shimizu et al., 2020). Consistent with these findings, we demonstrate here that anergy in tolerized T cells involves a membrane-proximal block in cell signaling because Zap70 and PKCθ fail to localize efficiently at the immune synapse, thus failing to induce AP-1 proteins efficiently. Furthermore, we show that the block can be largely bypassed using PMA to directly activate DAG-inducible pathways downstream of PKCθ and Ras (Figure 2F).

### The Inducible Gene-Regulatory Network Is Rewired in Tolerized T Cells

Our data establish that activation of repressive pathways during tolerization leads to loss of both epigenetic priming and inducible activation of many immune response genes. Our genome-wide analyses demonstrate that the TCR/CD28-inducible gene-regulatory network is rewired compared with both naive T cells and normal effector T cells after tolerization. It is likely that much of the epigenetic reprogramming described for T_B_ and memory T cells is either aborted or erased as part of the tolerization process. Indeed, tolerized cells successfully prime only 3% of the pDHSs defined by us previously in memory T cells.

The altered signaling network present in tolerized T cells activates different TF networks compared with the ones detected in naive T cells in both resting and activated cells. *Maf* and *Prdm1* levels were elevated in resting tolerant cells, and we observed much higher inducible activation of *Nfil3*, *Maf*, and *Prdm1* in tolerant cells than in naive T cells. *Maf* expression correlates directly with *Il10* expression in tolerized cells (Burton et al., 2014) and consistent with these findings, Gabryšová et al. (2018) have shown that c-Maf controls IL-10 production in Th1, Th2, and Th17 subsets. Here we show that induction of tolerance involves the opening of chromatin at known c-Maf binding sites (Figure 2), and that the tDHSs are enriched for AP-1 motifs where c-Maf is able to bind with other AP-1 proteins (Kataoka et al., 1994). Furthermore, NFIL3, c-Maf, and Blimp1 are all likely to be important drivers of anergy and immunosuppression because they were previously identified as regulators of *Il10*, *Havcr2*, *Tigit*, and/or *Pdcd1* expression in either T cells suppressed by IL-27 (Chihara et al., 2018, Zhu et al., 2015) or TILs derived from acute myeloid leukemia patients (Zhu et al., 2017). These observations were confirmed using regulatory T cells lacking Blimp1 (Cretney et al., 2018). Overexpression of c-Maf is partially sufficient to induce the exhausted T cell program, and *Maf*-deficient T cells are more efficient at targeting tumors *in vivo* (Giordano et al., 2015).

The gene-regulatory network that is activated in Ag-stimulated tolerant cells has diverged from that detected in naive T cells or T_B_ cells. By integrating data identifying the TF motifs present within the N_Ag_- or T_Ag_-specific iDHSs with protein expression levels and foot-printing analyses, we have been able to infer a model of how signaling to TFs is de-regulated during tolerization. This includes: (1) a suppression of signaling to AP-1 and NF-κB, (2) an increased utilization of NFAT in the absence of AP-1, and (3) an increased occupancy of composite AP-1/IRF elements. Indeed, increased binding of AP-1/IRF may occur through the suppression of certain AP-1 family members. The AP-1/IRF complex has been shown to contain BATF, JunD, and/or JunB, but not c-Fos (Glasmacher et al., 2012, Li et al., 2012). Although we observed a reduction in c-Fos, FosB, and JunB protein levels in T_Ag_ cells, the level of JunD remained stable, which is of interest here because JunD can function to suppress T cell responses (Meixner et al., 2004). The data also imply redistribution of AP-1 proteins away from conventional NFAT/AP-1 sites, which are typically bound by Jun/Fos heterodimers (Chen et al., 1998) and regulate immune response genes such as *CSF2* (Johnson et al., 2004), to AP-1/IRF sites found at immune-modulatory genes, such as *Il10* and *Nrp1*. By comparing our data with published data, we found that T_Ag_-specific DHSs were associated with a gain of BATF and IRF4 binding and a loss of JunB, which is consistent with our model. Furthermore, these same sets of TFs have been implicated in rewiring gene-regulatory networks in exhausted CD8 T cells ,where BATF, IRF4, and NFAT bound at the same sites at the immuno-regulatory genes *Lag3*, *Havcr2*, *Tigit*, *Ctla4*, and *Pdcd1* (Man et al., 2017), and in Tr1 cells, where IRF1 and BATF are key for preparing the chromatin landscape for induction of the Tr1 gene program (Kröger, 2017).

### The Tolerized State Shares Features with Anergic and Exhausted T Cells

Our previous genome-wide mRNA analyses (Burton et al., 2014) and the data described here demonstrate that tolerized T cells have moved away from the normal effector T cell program and closer to that of the anergic state. For example, 43% of the 1,033 tDHSs identified here in tolerized CD4 cells were also reprogrammed in a chronic LCMV (lymphocytic choriomeningitis virus) infection model of CD8 T cell exhaustion, including the *Ctla4* and *Il10* loci, whereas most of the epigenetic priming seen in memory T cells is absent (Sen et al., 2016). Tolerized cells resemble, but are not identical to, other anergic states, including exhausted T cells produced during chronic infections, exhausted TILs, T cells rendered anergic by unbalanced TCR/CD28 signaling, and *in vitro*-derived Tr1 cells (Figure S7). In each case a concerted program of inhibitory receptor gene expression is activated, which typically includes *Lag3*, *Havcr2* (TIM3), *Tigit*, *Ctla4*, and *Prdm1* (Blimp1) (Mognol et al., 2017, Singer et al., 2016, Wherry and Kurachi, 2015), which oppose the functions of co-activators such as CD28 (Chen and Flies, 2013). These anergic states also involve activation of a gene regulation network driven by NFAT binding independently of AP-1 (Martinez et al., 2015), and we find that certain AP-1 family proteins are downregulated in tolerized cells.

We also observed some differences between our *in vivo*-generated tolerant T cells and some other models of anergy. Studies of exhausted T cells from TILs (Mognol et al., 2017) and *in vitro*-derived tolerant T cells (Liu et al., 2019) found evidence that TCR signaling to NR4A family TFs is maintained, whereas we found that this pathway was suppressed in tolerized T cells. We also observed a loss of LEF/TCF consensus binding motifs in T_Ag_ DHSs, whereas TCF-1 plays an important role in controlling the differentiation of precursors that give rise to exhausted T cells during chronic infections (Chen et al., 2019). A direct comparison of mRNA data described above with two independent studies of anergic T cells suggests that tolerized cells, Tr1 cells, and CD8^+ve^ TILs all upregulate expression of TIM3, LAG3, Blimp1, and NFIL3. However, tolerant and Tr1 cells preferentially express Maf, IL-10, and IL-21, whereas tolerant T cells and TILs preferentially express PD-1 (Figure S7).

The sum of these studies reveals that different anergic and regulatory T cell states do indeed share the common utilization of immuno-suppressive factors by virtue of preferential epigenetic priming of their genes, thereby rendering them more responsive. The deficiency of the immune response is a direct consequence of inhibitory receptors blocking induction of most inducible genes. These studies also give encouragement for the expanded use of Ag-inducible tolerization as a strategy for combatting auto-immune disorders, and give clues as to how immune responses might be boosted in cancer and chronic infections. Significantly, this is no longer a distant goal, because peptide therapy is already being tested in auto-immune diseases. Our recent phase 1 and 2 clinical trials show that the MBP peptide tolerization strategy described above is giving positive results as a therapy for multiple sclerosis (Chataway et al., 2018), and a similar protocol using thyrotropin receptor peptides has delivered improvements in patients with Graves’ hyperthyroidism (Pearce et al., 2019). The current study has now provided a comprehensive understanding of the molecular basis of this therapeutic approach, which may one day become common practice.

## STAR★Methods

### Key Resources Table

**Table:** 

REAGENT or RESOURCE	SOURCE	IDENTIFIER
**Antibodies**		

anti-c-Jun Rabbit monoclonal	Cell Signaling Technologies	Cat# 9165; RRID: AB_2130165
anti-c-Fos Rabbit monoclonal	Cell Signaling Technologies	Cat# 2250; RRID: AB_2247211
anti-FosB Rabbit Monoclonal	Cell Signaling Technologies	Cat# 2251; RRID: AB_2106903
anti-JunB Rabbit Polyclonal	Santa Cruz Biotechnology	Cat# sc-46X; RRID: AB_2130022
anti-JunD Rabbit Polyclonal	Santa Cruz Biotechnology	Cat# sc-74X; RRID: AB_2130711
anti-Beta 2 Microglobulin.	Abcam	Cat# ab75853; RRID: AB_1523204
anti-PKCθ	BD BioScience	Cat# 610089; RRID:AB_397496
anti-PKCθ pT538 polyclonal	Cell Signaling Technologies	Cat# 9377; RRID:AB_2172071
biotinylated anti-CD3ε	BD BioScience	Cat# 553059; RRID:AB_394592
biotinylated anti-CD28	BD BioScience	Cat# 553296; RRID:AB_394765
anti-Zap70	Cell Signaling Technologies	Cat# 3165; RRID:AB_2218656
anti-CD28	Abcam	Cat# ab25234; RRID:AB_470416
anti-Cbl-b polyclonal H121	Santa Cruz	Cat# sc-8006; RRID: AB_2070711
goat anti-rabbit IgG-DyLight488	Jackson Immunoresearch	Cat# 305-486-006; RRID:AB_2339508
donkey anti-mouse IgG-Cy3	Jackson Immunoresearch	Cat# 715-165-150; RRID:AB_2340813

**Chemicals, Peptides, and Recombinant Proteins**		

MBP Ac1-9[4Y] 90% Purity (AcASQYRPSQR)	GL Biochem Shanghai	Custom product
Streptavidin	Jackson Immunoresearch	Cat# 016-000-084; RRID:AB_2337233
LPS	Sigma Aldrich	Cat# L8274
PFA	Electron Microscopy Sciences	Cat# 15710.
Murine rIL-12	Peprotech	Cat# 210-12P80H
Human rIL-2	R&D Systems	Cat# 202-IL
Murine rGM-CSF	Mitenyi Biotech	Cat# 130-094-043
DNase I	Worthington	DPPF Grade
Calcium Ionophore A23187	Sigma Aldrich	Cat# C7522
Phorbol 12-myristate 13-acetate	Sigma Aldrich	Cat# P8139

**Critical Commercial Assays**		

MagniSort Mouse CD4 T cell Enrichment Kit	Thermofisher	Cat# 8804-6821-74
CD4+ T cell Isolation Kit II	Miltenyi Biotech	Cat# 130-095-248
PicoPure RNA Isolation Kit	Thermofisher	Cat# KIT0204
Superscript IV reverse transcriptase	Thermofisher	Cat# 18090010
Applied Biosystems SYBR green master mix	Thermofisher	Cat# 4309155
NEBNext rRNA depletion kit	NEB	Cat# E6310L
NEBNext Ultra II kit	NEB	Cat# E7760S
NEBNext oligonucleotides	NEB	Cat# E7335S
NextSeq® High Output kit v2.5 150 cycles	Illumina	Cat# 20024907
NextSeq® 500/550 High Output kit v2 75 cycles	llumina	Cat# FC 404-2005
Kapa Hyper-Prep kit	Kapa Biosystems	Cat# KK8500
Kapa Library Quantification Kit	Kapa Biosystems	Cat# KK4824
MinElute Gel Extraction Kit	QIAGEN	Cat# 2860

**Deposited Data**		

Raw and analyzed data	This paper	GEO: GSE147268
TCF1 ChIP - thymocytes	(Dose et al., 2014)	GEO: GSE46662
c-MAF ChIP-seq - Th17 cells	(Ciofani et al., 2012)	GEO: GSM1004799
NFAT-CA-RIT-NFAT1, mock NFAT1, NFAT-CA-RIT-NFAT1 PI, mock NFAT1 PI in CD8 T cells	(Martinez et al., 2015)	GEO: GSM1570758
IRF4 ChIP-seq in CD4 T cells and BATF in CD4 +IL-21 T cells	(Li et al., 2012)	GEO: GSE39756
JUNB ChIP-seq in CD4 T_B_ PI cells, H3K4me2 and H3K27ac ChIP-seq in CD4 T_B_ and T_B_ PI cells, and DNase I in CD4 T_B_ and CD4 T_B_ PI	(Bevington et al., 2016)	GEO: GSE67443
p65 ChIP-seq in Tconv cells stimulated with CD3/CD28	(Oh et al., 2017)	GEO: GSE99319

**Experimental Models: Organisms/Strains**		

Mouse: Tg4-H2^u^	(Liu et al., 1995)	https://pubmed.ncbi.nlm.nih.gov/7584132
Mouse: B10.PL mice (B10.PL-*H2*^*u*^*H2-T18*^*a*^/(73NS) SnJ	Jackson Laboratory	

**Oligonucleotides**		

Oligonucletide primers are listed in Table S6		N/A

**Software and Algorithms**		

TopHat (version 2.1.1)	(Kim et al., 2013)	https://ccb.jhu.edu/software/tophat/index.shtml
HTSeq-count (version 0.9.1)	(Anders et al., 2015)	https://pypi.org/project/HTSeq/
DESeq2 (version 1.18)	(Love et al., 2014)	https://bioconductor.org/packages/release/bioc/html/DESeq2.html
EdgeR (version 3.24)	(Robinson et al., 2010)	https://bioconductor.org/packages/release/bioc/html/edgeR.html
R (version 3.4.3)	The R project for Statistical Computing	https://www.r-project.org/
Bowtie2 v2.2.3	(Langmead and Salzberg, 2012)	http://bowtie-bio.sourceforge.net/bowtie2/index.shtml
MACs version 1.4.2	(Zhang et al., 2008)	https://github.com/taoliu/MACS
MACS2 callpeak (Galaxy Version 2.1.1)	(Zhang et al., 2008)	https://usegalaxy.org/
HOMER v4.9.1	(Heinz et al., 2010)	http://homer.ucsd.edu/homer/index.html
Java Treeview v1.1	(Saldanha, 2004)	https://sourceforge.net/projects/jtreeview/
Bedtools (Galaxy Version 2.26.0.0)	(Quinlan and Hall, 2010) .	https://bedtools.readthedocs.io/en/latest/
Trimmomatic v0.32	(Bolger et al., 2014)	http://www.usadellab.org/cms/index.php?page=trimmomatic
Wellington Method - pyDNase 0.2.4	(Piper et al., 2013)	https://pythonhosted.org/pyDNase/

### Resource Availability

#### Lead Contact

Further information and requests for resources and reagents should be directed to and will be fulfilled by the Lead Contact Peter Cockerill (p.n.cockerill@bham.ac.uk).

#### Material Availability

This study did not generate any unique reagents.

#### Data and Code Availability

The accession number for the data reported in this paper is GEO: GSE147268. The article also includes previously published datasets**:** TCF1 ChIP-Seq in thymocytes – GSE46662 (Dose et al., 2014), c-MAF ChIP-seq in Th17 cells - GEO: GSM1004799 (Ciofani et al., 2012), NFAT-CA-RIT-NFAT1, WT NFAT1, NFAT-CA-RIT-NFAT1 PI, WT NFAT1 PI in CD8 T cells - GEO: GSM1570758 (Martinez et al., 2015). IRF4 ChIP-seq in CD4 T cells and BATF in CD4 +IL-21 T cells GEO: GSE39756 (Li et al., 2012), JUNB ChIP-seq in CD4 T_B_ PI cells, H3K4me2 and H3K27ac ChIP-seq in CD4 T_B_ and T_B_ PI cells, and DNase I in CD4 T_B_ and CD4 T_B_ PI, GEO: GSE67443 (Bevington et al., 2016) and p65 ChIP-seq in Tconv cells stimulated with CD3/CD28 - GEO: GSE99319 (Oh et al., 2017).

### Experimental Model and Subject Details

#### Tg4 transgenic Mice

Tg4-H2^u^ mice expressing the αβ TCR (Vα4, Vβ8.2) of the MBP Ac1-9-specific hybridoma 1934.4, derived from an encephalitogenic T cell clone have been described previously (Liu et al., 1995). Tg4 mice have a skewed CD4^+^ T cell repertoire where over 90% of the CD4^+^ T cells are Vβ8^+^. Male and female mice aged between 6-12 weeks were used. Animals were housed under specific pathogen-free conditions, and experiments were performed in accordance with the UK Home Office Project License held by D.C.W. and approved by the University of Birmingham ethical review committee. T cells from the peripheral blood of Tg4 mice were phenotyped for CD4 and Vβ8 by flow cytometry.

#### Cells

Th1 effector cells were generated *in vitro* by culturing splenocytes, from which red blood cells were depleted, from Tg4 mice with 10 μg/ml MBP Ac1-9 [4K] and 5 ng/ml rmIL-12 (Peprotech) for 72 hours in complete RPMI. Cells were further expanded for 6-8 days in complete RPMI supplemented with 20 U/ml rhIL-2 (R&D) (Hill et al., 2013). Splenocytes from tolerized mice were also expanded *in vitro* before analysis by culturing for 5 days in complete RPMI with 10 μg/ml MBP Ac1-9 [4K] and 20 U/ml rhIL-2 (Anderson et al., 2005). Bone marrow-derived dendritic cells were generated from B10.PL mice (B10.PL-*H2*^*u*^ *H2-T18*^*a*^/(73NS) SnJ originally from the Jackson Laboratory) by culturing the non-adherent fraction of disaggregated bone marrow with 10 ng/ml rmGM-CSF (Miltenyi Biotec) in complete RPMI for 10-12 days (Inaba et al., 1992). BM-DC were routinely > 85% MHC-II^+^ as measured by flow cytometry.

### Method Details

#### Induction of tolerance by sub-cutaneous injection of peptides

For most of the tolerized T cell experiments described here (T_0_ and T_Ag_), tolerance was induced by administering 200 μL doses of the MBP Ac1-9[4Y] subcutaneously in the flank of un-anaesthetized mice every 3-4 days for 7 doses. The amount of peptide was increased until the maximum dose was reached and maintained, as defined in Figure 1C (0.08 μg, 0.8 μg, 8 μg, 80 μg peptide/0.2ml PBS). For N_0_, 200 μL of PBS was administered in the same dosing strategy. 2 hours prior to sacrifice a final dose of 200 μL of PBS (N_0_ and T_0_) or 200 μL of 400 μg/ml MBP Ac1-9 [4Y] (N_Ag_ and T_Ag_) was given. For T_0M,_ mice were sacrificed 3 weeks after the final dose.

#### Induction of tolerance by intra-nasal peptides

In Figures 6A–6G and S6, Tg4 mice were tolerized by intranasal administration of 10 doses of 80 μg MBP Ac1-9 [4Y]) at 3-4 day intervals (Gabrysová et al., 2009, Sundstedt et al., 2003).

#### CD4+ T cell Selection

For most experiments CD4^+^ T cells were purified using the MagniSort Mouse CD4 T cell Enrichment Kit (8804-6821-74) according to the manufacturer’s instructions. For the western blotting in Figure 6E and microscopy experiments Figures 6 and S6, CD4^+^ T cells were purified by magnetic separation (Miltenyi Isolation Kit II) according to the manufacturer’s instruction.

#### *In vitro* activation of T cells

For western blot analyses, CD4^+^ T cells were activated by incubating with 10 μg/ml biotinylated anti-CD3ε (clone 145-2C11, BD**)** and 10 μg/ml biotinylated anti-CD28 (clone 37.51, BD) for 30 minutes followed by crosslinking with 10 μg/ml streptavidin (Jackson Immunoresearch) at 37°C for the indicated times. For mRNA and DNase-Seq analyses, CD4+ T cells were re-suspended in complete RPMI with 20 ng/ml phorbol myristate acetate (PMA) and 2 μM Calcium Ionophore A23187 (CaI) (N_PI_ and T_PI_). For the mRNA time course cells were harvested at 30’, 60’, 120’, 240’ and 480’. For DNase-Seq and RNA-seq experiments cell were harvested at 120’.

#### Western blot analyses of protein expression

Proteins were extracted in 50 mM Tris, 120 mM NaCl, 1 mM EDTA with 1% IGEPAL CA-630 and protease and phosphatase inhibitor cocktails (Thermo) before SDS-PAGE and western blotting. The antibodies used in this study were anti-PKCθ clone 27/PKCθ (BD Bioscience), anti-PKCθ pT538 ployclonal (Cell Signaling Technologies #9377) , anti-c-Jun Rabbit monoclonal (Cell Signaling Technologies #9165), anti-c-fos Rabbit monoclonal (Cell Signaling Technologies #2250), anti-fosB Rabbit monoclonal (Cell Signaling Technologies #2251), anti-JunB Rabbit polyclonal (Santa Cruz Biotehnology #sc-46X) anti-JunD Rabbit polyclonal (Santa Cruz Biotehnology #sc-74X) and anti-beta-2-microglobulin (Abcam #AB75853).

#### Microscopy sample preparation

Before imaging, BM-DC were incubated with 1 μg/ml LPS (Sigma) for 16-18 hours, washed and then incubated for 2 hours with 1 μg/ml MBP Ac1-9[4Y]. DC-T cell couples for confocal microscopy were prepared by combining activated and peptide-loaded BM-DC and magnetically enriched CD4+ T cell at a ratio of 1:2 at 3x10^6^ cells/ml. Coupling was synchronized by brief centrifugation (75 *g*) in a round bottom plate and the cells were allowed to interact for 5 minutes at 37°C before the cells were gently transferred to pre-warmed glass slides and incubated for a further 5 minutes. Cells were fixed in 4% buffered PFA before immune-labeling. For TIRF-M imaging, glass coverslips (Mat-Tek II) were pre-coated with 2 μg/ml anti-CD3ε clone 145-2C11, BD**)** and 1 μg/ml anti-CD28 (clone 37.51, BD). CD4^+^ T cells were allowed to interact with the antibody coated surface for 8 minutes at 37°C before fixation and immune-labeling. Primary antibodies used were anti-PKCθ clone 27/PKCθ (BD Bioscience) at 2.5 μg/ml, anti-Zap70 clone D1C10E (Cell Signaling Technologies) at 2.5 μg/ml, anti-CD28 clone PV.1 (Abcam) at 1 μg/ml and anti-Cbl-b polyclonal H121 (Santa Cruz - discontinued) at 5 μg/ml. Secondary antibodies, goat anti-rabbit IgG-DyLight488, donkey anti-mouse IgG-Cy3 and goat anti-Armenian hamster-Cy2. (Jackson Immunoresearch) were used at 2.5 μg/ml.

#### Microscopy

Confocal laser scanning microscopy was performed on a DMI 6000 inverted epifluorescence microscope equipped with an SP5-AOBS confocal scanning system (Leica Microsystems). Total internal reflection fluorescence microscopy (TIRF-M) was performed on a DMI 6000 inverted epifluorescence microscope attached to an AM TIRF MC system (Leica). An estimated penetration depth of 100 nm was used. Multi-color imaging was performed using sequential laser scanning. Conjugates between APC and T cells were identified using the bright field channel without reference to fluorescence channels and laser power, gain, offset and averaging/additive functions were kept constant between samples which were to be compared. Image analysis was performed with Volocity 5 (Perkin Elmer). Analysis of synaptic protein enrichment in DC-T cell couples were performed by measuring the fluorescence intensity of the T cell membrane-proximal region at areas of the interface and distal from the interface. The enrichment of a protein at the interface was represented as the ratio if these values. In TIRF-M experiments, protein accumulation at the interface was assessed by measuring the relative fluorescence intensity within the cell footprint, defined by bright field images. Where indicated, the ratio of two fluorescence channels is presented in pseudo color using the ‘Ratio’ tool in Volocity 5.

#### mRNA isolation and quantitative PCR

Total RNA was isolated using PicoPure RNA Isolation Kit (KIT0204) according to the manufacturer’s instructions. RNA was reverse transcribed using Superscript IV reverse transcriptase (Thermo Fisher) according to the manufacturer’s protocol. qPCR was carried out using Applied Biosystems SYBR green master mix (Thermo Fisher). Primers used for qPCR are listed in Table S6.

#### RNA-seq library preparation

Ribosomal RNA was depleted using NEBNext rRNA depletion kit (E6310L) according to the manufacturer’s instructions. RNA-seq libraries were prepared from 30 ng RNA using the NEBNext Ultra II kit (E7760S) using the NEBNext oligonucleotides (E7335S) according to the manufacturer’s instructions. Samples were sequenced using the NextSeq® High Output kit v2.5 150 cycles (Illumina, 20024907).

#### DNase I hypersensitive site analysis

DNase I digestions were carried out as described previously (Bevington et al., 2016). In brief, cells were re-suspended at 1x10^7^ cells/ml in DNase I buffer (60 mM KCl, 15 mM NaCl, 5 mM MgCl_2_, 100 mM Tris-HCl pH 7.4, 1 mM EGTA pH 7.4, 0.3 M sucrose, 0.2% NP40, 1 mM CaCl_2_ and DNase I). 1x10^6^ cells were digested for 3′ at 22°C before the reaction was terminated by the addition of SDS (final concentration 0.5%). Samples were treated with 0.5 mg/ml Proteinase K at 37°C overnight followed by 0.2 mg/ml RNase A for 1 hour. DNA was purified by phenol/chloroform extraction and fragments were separated by agarose gel electrophoresis. Small DNA fragments (50-250 bp) were extracted and purified (QIAGEN Minelute Gel extraction Kit) before library preparation.

#### Library preparation

DNase I libraries were prepared using the Kapa Hyper-Prep kit (KAPA Biosystems) according the manufacturer’s instructions. Libraries were amplified by PCR and fragments of 200-300bp were gel purified (QIAGEN Minelute Gel extraction Kit) before qPCR validation and quantification (KAPA Library Quantification Complete kit -ABI Prism®). Samples were pooled and sequenced using the NextSeq® 500/550 High Output kit v2 75 cycles (Illumina, FC 404-2005).

### Quantification and Statistical Analysis

#### RNA-seq Analysis

Paired-end reads were aligned to the mouse genome (version mm9, build 37) using TopHat (version 2.1.1) (Kim et al., 2013, Kim and Salzberg, 2011, Langmead et al., 2009, Trapnell et al., 2009). Transcript counts were calculated with HTSeq-count (version 0.9.1) (Anders et al., 2015) using gene models from Ensembl as reference. Differential gene expression analysis was carried out using the DESeq2 (version 1.18) (Love et al., 2014) and EdgeR (version 3.24) (McCarthy et al., 2012, Robinson et al., 2010) packages in R (version 3.4.3). Only genes that could be reproducibly identified as statistically significant by both DESeq2 (FDR < 0.05) and EdgeR (p < 0.05) were retained for further analysis. This was done to reduce the number of false positive results and ensure that the gene sets used in downstream analyses were robust. Genes that had a fold-change of at least 2 (as calculated by DESeq2) were deemed to be differentially expressed. PCA and heatmap plots were generated using ggplot in R.

#### Definition of gene expression groups

The 460 T_0_ and 80 N_0_ specific gene mRNAs were defined as 2-fold upregulated between T_0_ and N_0_ and N_0_ and T_0_ respectively with a minimum read count of 50. The 138 T_Ag_ and 226 N_Ag_ specific genes were defined as 2 fold upregulated between T_Ag_ and N_Ag_ and N_Ag_ and T_Ag_ respectively with a minimum read count and 50. In addition these genes were filtered to only include genes which were 2 fold upregulated when T_0_ or N_0_ were stimulated with Ag.

#### DNase-Seq - alignment, coverage and peak detection

Raw sequencing reads were aligned to mm9 using *bowtie2* (Galaxy Version 2.3.2.2) (Langmead and Salzberg, 2012) with the preset –very-sensitive-local. Coverage files were generated using MACs version 1.4.2 using –g mm –keep-dup auto –w -S as parameters (Zhang et al., 2008).

#### Normalization of DNase-Seq datasets

The BAM files from the duplicate samples of N_0_, T_0_, N_Ag_ and T_Ag_ were merged using BamTools (Galaxy Version 0.0.2). A master set of peaks were determined using MACS2 callpeak (Galaxy Version 2.1.1). 69743 summits were identified across all samples. The sequence tags ± 200bp from the peak summit were counted for the 69743 peaks for each individual sample using the annotatePeaks function of the HOMER package (Heinz et al., 2010). The samples were normalized using a correction factor based on the median of the top 25,000 peaks for each sample. Correction factors were then used to normalize genome browser scales, average sequence tag density plots and contrast levels in heatmaps.

#### Unions of DNase-Seq data

For each sample the sequence tags were counted ± 200bp for the 69743 peaks and the significant peaks were determined using a cut off which excluded background and insignificant peaks. Two samples were compared by merging the peaks using the sort and merge function of the bedtools package (Quinlan and Hall, 2010)to give a union which included common and unique peaks. For the unions between T_0_ and N_0_ peaks were further filtered to eliminate sine elements. The sequence tags were counted ± 200bp from the summits of the peaks in the union, normalized using the correction factor and the fold change difference was calculated between the 2 samples. The data were visualized as sequence tag density profiles ordered according to the fold change difference in sequence tag density of one sample compared to the other.

#### Sequence tag density profiles

Sequence tag density profiles were generated using the annotatePeaks function of the HOMER package using -hist 10 -ghist -size 2000 as parameters. Images were generated using Java Treeview v1.1 (Saldanha, 2004).

#### Average sequence tag density plots

Average sequence tag density plots ± 1 kb around the DHS summit were generated using the annotatePeaks function of the HOMER package using -hist 10 -size 2000 as parameters.

#### Differential peak analysis using DESeq2

Only peaks which were present in both samples for each condition were used in analyses. Reads were counted using featurecounts and DESeq2 (Galaxy version 2.11.40.6) was used to determine differential peaks between 2 conditions. Only regions with p < 0.05 were included.

#### RNA fold change heatmaps

Peaks were assigned to the closest gene within 100 kb of the transcription start site (TSS) using the annotatePeaks.pl function in Homer v4.9.1 (Heinz et al., 2010). The fold-change values for these genes were then plotted as a heatmap using Java TreeView v1.1 (Saldanha, 2004).

#### Distance analyses

The distance between the DHSs and the TSSs of upregulated genes was calculated using the BEDTools closestBed function (Galaxy Version 2.26.0.0). Genes were grouped according to distance from the DHSs to the TSSs of the genes in intervals.

#### Motif discovery

*De novo* motif analysis was performed using the findMotifsGenome.pl function of the HOMER package. Motifs were identified ± 100 bp from the peak summit.

#### Footprinting analysis

Raw sequencing data from high-depth DNase-Seq experiments were processed to remove low-quality reads and sequencing adaptors with Trimmomatic v0.32 (Bolger et al., 2014). The processed reads were then aligned to the mouse genome (version mm9) with Bowtie2 v2.2.3 (Langmead and Salzberg, 2012) using the option–very-sensitive-local. Footprints were identified using the Wellington method implemented in pyDNase 0.2.4 (Piper et al., 2013). DNase I cut profiles were calculated using the in-built functions in pyDNase.

## Supplemental Information

Document S1. Figures S1–S7 and Table S6

Table S1. Normalized Read Counts for All Expressed Genes, Related to Figures 1 and S1

Table S2. Differentially Regulated Genes between T_0_ and N_0_, Related to Figures 1 and S1

Table S3. Differentially Regulated Genes between T_Ag_ and N_Ag_, Related to Figures 1 and S1

Table S4. T_0_-Specific Genes in Common with Tr1 Cells and CD^+ve^ TILs, Related to Figures 1 and S7

Table S5. T_0_-, N_0_-, T_Ag_-, and N_Ag_-Specific Gene Subsets, Related to Figures 2 and S2

Data S1. DNA-Seq Data with Normalized Read Counts for All Peaks and for the T_0_-, N_0_-, T_Ag_-, and N_Ag_-Specific Peak Subsets, Related to Figures 2, 3, 4, and 5

Document S2. Article plus Supplemental Information

## References

[bib1] Anders S., Pyl P.T., Huber W. (2015). HTSeq—a Python framework to work with high-throughput sequencing data. Bioinformatics.

[bib2] Anderson P.O., Sundstedt A., Yazici Z., Minaee S., O’Neill E.J., Woolf R., Nicolson K., Whitley N., Li L., Li S. (2005). IL-2 overcomes the unresponsiveness but fails to reverse the regulatory function of antigen-induced T regulatory cells. J. Immunol..

[bib3] Anderson A.C., Joller N., Kuchroo V.K. (2016). Lag-3, Tim-3, and TIGIT: Co-inhibitory Receptors with Specialized Functions in Immune Regulation. Immunity.

[bib4] Bachmaier K., Krawczyk C., Kozieradzki I., Kong Y.Y., Sasaki T., Oliveira-dos-Santos A., Mariathasan S., Bouchard D., Wakeham A., Itie A. (2000). Negative regulation of lymphocyte activation and autoimmunity by the molecular adaptor Cbl-b. Nature.

[bib5] Bevington S.L., Cauchy P., Piper J., Bertrand E., Lalli N., Jarvis R.C., Gilding L.N., Ott S., Bonifer C., Cockerill P.N. (2016). Inducible chromatin priming is associated with the establishment of immunological memory in T cells. EMBO J..

[bib6] Bevington S.L., Cauchy P., Cockerill P.N. (2017). Chromatin priming elements establish immunological memory in T cells without activating transcription: T cell memory is maintained by DNA elements which stably prime inducible genes without activating steady state transcription. BioEssays.

[bib7] Bolger A.M., Lohse M., Usadel B. (2014). Trimmomatic: a flexible trimmer for Illumina sequence data. Bioinformatics.

[bib8] Brignall R., Cauchy P., Bevington S.L., Gorman B., Pisco A.O., Bagnall J., Boddington C., Rowe W., England H., Rich K. (2017). Integration of Kinase and Calcium Signaling at the Level of Chromatin Underlies Inducible Gene Activation in T Cells. J. Immunol..

[bib9] Burkhart C., Liu G.Y., Anderton S.M., Metzler B., Wraith D.C. (1999). Peptide-induced T cell regulation of experimental autoimmune encephalomyelitis: a role for IL-10. Int. Immunol..

[bib10] Burton B.R., Britton G.J., Fang H., Verhagen J., Smithers B., Sabatos-Peyton C.A., Carney L.J., Gough J., Strobel S., Wraith D.C. (2014). Sequential transcriptional changes dictate safe and effective antigen-specific immunotherapy. Nat. Commun..

[bib11] Chataway J., Martin K., Barrell K., Sharrack B., Stolt P., Wraith D.C., ATX-MS1467 Study Group (2018). Effects of ATX-MS-1467 immunotherapy over 16 weeks in relapsing multiple sclerosis. Neurology.

[bib12] Chen L., Flies D.B. (2013). Molecular mechanisms of T cell co-stimulation and co-inhibition. Nat. Rev. Immunol..

[bib13] Chen L., Glover J.N., Hogan P.G., Rao A., Harrison S.C. (1998). Structure of the DNA-binding domains from NFAT, Fos and Jun bound specifically to DNA. Nature.

[bib14] Chen Z., Ji Z., Ngiow S.F., Manne S., Cai Z., Huang A.C., Johnson J., Staupe R.P., Bengsch B., Xu C. (2019). TCF-1-Centered Transcriptional Network Drives an Effector versus Exhausted CD8 T Cell-Fate Decision. Immunity.

[bib15] Chihara N., Madi A., Kondo T., Zhang H., Acharya N., Singer M., Nyman J., Marjanovic N.D., Kowalczyk M.S., Wang C. (2018). Induction and transcriptional regulation of the co-inhibitory gene module in T cells. Nature.

[bib16] Ciofani M., Madar A., Galan C., Sellars M., Mace K., Pauli F., Agarwal A., Huang W., Parkhurst C.N., Muratet M. (2012). A validated regulatory network for Th17 cell specification. Cell.

[bib17] Clemente-Casares X., Blanco J., Ambalavanan P., Yamanouchi J., Singha S., Fandos C., Tsai S., Wang J., Garabatos N., Izquierdo C. (2016). Expanding antigen-specific regulatory networks to treat autoimmunity. Nature.

[bib18] Cretney E., Leung P.S., Trezise S., Newman D.M., Rankin L.C., Teh C.E., Putoczki T.L., Gray D.H., Belz G.T., Mielke L.A. (2018). Characterization of Blimp-1 function in effector regulatory T cells. J. Autoimmun..

[bib19] Dose M., Emmanuel A.O., Chaumeil J., Zhang J., Sun T., Germar K., Aghajani K., Davis E.M., Keerthivasan S., Bredemeyer A.L. (2014). β-Catenin induces T-cell transformation by promoting genomic instability. Proc. Natl. Acad. Sci. USA.

[bib20] Fang D., Liu Y.C. (2001). Proteolysis-independent regulation of PI3K by Cbl-b-mediated ubiquitination in T cells. Nat. Immunol..

[bib21] Fujiwara M., Anstadt E.J., Clark R.B. (2017). Cbl-b Deficiency Mediates Resistance to Programmed Death-Ligand 1/Programmed Death-1 Regulation. Front. Immunol..

[bib22] Gabrysová L., Nicolson K.S., Streeter H.B., Verhagen J., Sabatos-Peyton C.A., Morgan D.J., Wraith D.C. (2009). Negative feedback control of the autoimmune response through antigen-induced differentiation of IL-10-secreting Th1 cells. J. Exp. Med..

[bib23] Gabryšová L., Alvarez-Martinez M., Luisier R., Cox L.S., Sodenkamp J., Hosking C., Pérez-Mazliah D., Whicher C., Kannan Y., Potempa K. (2018). c-Maf controls immune responses by regulating disease-specific gene networks and repressing IL-2 in CD4^+^ T cells. Nat. Immunol..

[bib24] Giordano M., Henin C., Maurizio J., Imbratta C., Bourdely P., Buferne M., Baitsch L., Vanhille L., Sieweke M.H., Speiser D.E. (2015). Molecular profiling of CD8 T cells in autochthonous melanoma identifies Maf as driver of exhaustion. EMBO J..

[bib25] Glasmacher E., Agrawal S., Chang A.B., Murphy T.L., Zeng W., Vander Lugt B., Khan A.A., Ciofani M., Spooner C.J., Rutz S. (2012). A genomic regulatory element that directs assembly and function of immune-specific AP-1-IRF complexes. Science.

[bib26] Heinz S., Benner C., Spann N., Bertolino E., Lin Y.C., Laslo P., Cheng J.X., Murre C., Singh H., Glass C.K. (2010). Simple combinations of lineage-determining transcription factors prime cis-regulatory elements required for macrophage and B cell identities. Mol. Cell.

[bib27] Heissmeyer V., Macián F., Im S.H., Varma R., Feske S., Venuprasad K., Gu H., Liu Y.C., Dustin M.L., Rao A. (2004). Calcineurin imposes T cell unresponsiveness through targeted proteolysis of signaling proteins. Nat. Immunol..

[bib28] Hill E.V., Oakley C.M., Ng T.H.S., Burton B.R., Wraith D.C. (2013). Glycogen synthase kinase-3 regulates IL-10 production in Th1 cells. Immunology.

[bib29] Inaba K., Inaba M., Romani N., Aya H., Deguchi M., Ikehara S., Muramatsu S., Steinman R.M. (1992). Generation of large numbers of dendritic cells from mouse bone marrow cultures supplemented with granulocyte/macrophage colony-stimulating factor. J. Exp. Med..

[bib30] Jeon M.S., Atfield A., Venuprasad K., Krawczyk C., Sarao R., Elly C., Yang C., Arya S., Bachmaier K., Su L. (2004). Essential role of the E3 ubiquitin ligase Cbl-b in T cell anergy induction. Immunity.

[bib31] Johnson B.V., Bert A.G., Ryan G.R., Condina A., Cockerill P.N. (2004). Granulocyte-macrophage colony-stimulating factor enhancer activation requires cooperation between NFAT and AP-1 elements and is associated with extensive nucleosome reorganization. Mol. Cell. Biol..

[bib32] Karwacz K., Miraldi E.R., Pokrovskii M., Madi A., Yosef N., Wortman I., Chen X., Watters A., Carriero N., Awasthi A. (2017). Critical role of IRF1 and BATF in forming chromatin landscape during type 1 regulatory cell differentiation. Nat. Immunol..

[bib33] Kataoka K., Noda M., Nishizawa M. (1994). Maf nuclear oncoprotein recognizes sequences related to an AP-1 site and forms heterodimers with both Fos and Jun. Mol. Cell. Biol..

[bib34] Kim D., Salzberg S.L. (2011). TopHat-Fusion: an algorithm for discovery of novel fusion transcripts. Genome Biol..

[bib35] Kim D., Pertea G., Trapnell C., Pimentel H., Kelley R., Salzberg S.L. (2013). TopHat2: accurate alignment of transcriptomes in the presence of insertions, deletions and gene fusions. Genome Biol..

[bib36] Kröger A. (2017). IRFs as competing pioneers in T-cell differentiation. Cell. Mol. Immunol..

[bib37] Langmead B., Salzberg S.L. (2012). Fast gapped-read alignment with Bowtie 2. Nat. Methods.

[bib38] Langmead B., Trapnell C., Pop M., Salzberg S.L. (2009). Ultrafast and memory-efficient alignment of short DNA sequences to the human genome. Genome Biol..

[bib39] Li D., Gál I., Vermes C., Alegre M.L., Chong A.S., Chen L., Shao Q., Adarichev V., Xu X., Koreny T. (2004). Cutting edge: Cbl-b: one of the key molecules tuning CD28- and CTLA-4-mediated T cell costimulation. J. Immunol..

[bib40] Li P., Spolski R., Liao W., Wang L., Murphy T.L., Murphy K.M., Leonard W.J. (2012). BATF-JUN is critical for IRF4-mediated transcription in T cells. Nature.

[bib41] Li X., Gong L., Gu H. (2019). Regulation of immune system development and function by Cbl-mediated ubiquitination. Immunol. Rev..

[bib42] Liu G.Y., Fairchild P.J., Smith R.M., Prowle J.R., Kioussis D., Wraith D.C. (1995). Low avidity recognition of self-antigen by T cells permits escape from central tolerance. Immunity.

[bib43] Liu X., Wang Y., Lu H., Li J., Yan X., Xiao M., Hao J., Alekseev A., Khong H., Chen T. (2019). Genome-wide analysis identifies NR4A1 as a key mediator of T cell dysfunction. Nature.

[bib44] Love M.I., Huber W., Anders S. (2014). Moderated estimation of fold change and dispersion for RNA-seq data with DESeq2. Genome Biol..

[bib45] Macián F., García-Cózar F., Im S.H., Horton H.F., Byrne M.C., Rao A. (2002). Transcriptional mechanisms underlying lymphocyte tolerance. Cell.

[bib46] Man K., Gabriel S.S., Liao Y., Gloury R., Preston S., Henstridge D.C., Pellegrini M., Zehn D., Berberich-Siebelt F., Febbraio M.A. (2017). Transcription Factor IRF4 Promotes CD8+ T Cell Exhaustion and Limits the Development of Memory-like T Cells during Chronic Infection. Immunity.

[bib47] Martinez G.J., Pereira R.M., Äijö T., Kim E.Y., Marangoni F., Pipkin M.E., Togher S., Heissmeyer V., Zhang Y.C., Crotty S. (2015). The transcription factor NFAT promotes exhaustion of activated CD8^+^ T cells. Immunity.

[bib48] Mayo L., Cunha A.P., Madi A., Beynon V., Yang Z., Alvarez J.I., Prat A., Sobel R.A., Kobzik L., Lassmann H. (2016). IL-10-dependent Tr1 cells attenuate astrocyte activation and ameliorate chronic central nervous system inflammation. Brain.

[bib49] McCarthy D.J., Chen Y., Smyth G.K. (2012). Differential expression analysis of multifactor RNA-Seq experiments with respect to biological variation. Nucleic Acids Res..

[bib50] Meixner A., Karreth F., Kenner L., Wagner E.F. (2004). JunD regulates lymphocyte proliferation and T helper cell cytokine expression. EMBO J..

[bib51] Mognol G.P., Spreafico R., Wong V., Scott-Browne J.P., Togher S., Hoffmann A., Hogan P.G., Rao A., Trifari S. (2017). Exhaustion-associated regulatory regions in CD8^+^ tumor-infiltrating T cells. Proc. Natl. Acad. Sci. USA.

[bib52] O’Garra A., Vieira P. (2007). T(H)1 cells control themselves by producing interleukin-10. Nat. Rev. Immunol..

[bib53] Oh H., Grinberg-Bleyer Y., Liao W., Maloney D., Wang P., Wu Z., Wang J., Bhatt D.M., Heise N., Schmid R.M. (2017). An NF-κB Transcription-Factor-Dependent Lineage-Specific Transcriptional Program Promotes Regulatory T Cell Identity and Function. Immunity.

[bib54] O’Neill E.J., Day M.J., Wraith D.C. (2006). IL-10 is essential for disease protection following intranasal peptide administration in the C57BL/6 model of EAE. J. Neuroimmunol..

[bib55] Pearce S.H.S., Dayan C., Wraith D.C., Barrell K., Olive N., Jansson L., Walker-Smith T., Carnegie C., Martin K.F., Boelaert K. (2019). Antigen-Specific Immunotherapy with Thyrotropin Receptor Peptides in Graves’ Hyperthyroidism: A Phase I Study. Thyroid.

[bib56] Piper J., Elze M.C., Cauchy P., Cockerill P.N., Bonifer C., Ott S. (2013). Wellington: a novel method for the accurate identification of digital genomic footprints from DNase-seq data. Nucleic Acids Res..

[bib57] Pot C., Jin H., Awasthi A., Liu S.M., Lai C.Y., Madan R., Sharpe A.H., Karp C.L., Miaw S.C., Ho I.C., Kuchroo V.K. (2009). Cutting edge: IL-27 induces the transcription factor c-Maf, cytokine IL-21, and the costimulatory receptor ICOS that coordinately act together to promote differentiation of IL-10-producing Tr1 cells. J. Immunol..

[bib58] Qiao G., Zhao Y., Li Z., Tang P.Q., Langdon W.Y., Yang T., Zhang J. (2013). T cell activation threshold regulated by E3 ubiquitin ligase Cbl-b determines fate of inducible regulatory T cells. J. Immunol..

[bib59] Quinlan A.R., Hall I.M. (2010). BEDTools: a flexible suite of utilities for comparing genomic features. Bioinformatics.

[bib60] Robinson M.D., McCarthy D.J., Smyth G.K. (2010). edgeR: a Bioconductor package for differential expression analysis of digital gene expression data. Bioinformatics.

[bib61] Roncarolo M.G., Gregori S., Bacchetta R., Battaglia M. (2014). Tr1 cells and the counter-regulation of immunity: natural mechanisms and therapeutic applications. Curr. Top. Microbiol. Immunol..

[bib62] Sabatos-Peyton C.A., Verhagen J., Wraith D.C. (2010). Antigen-specific immunotherapy of autoimmune and allergic diseases. Curr. Opin. Immunol..

[bib63] Saldanha A.J. (2004). Java Treeview—extensible visualization of microarray data. Bioinformatics.

[bib64] Schwartz R.H. (2003). T cell anergy. Annu. Rev. Immunol..

[bib65] Sen D.R., Kaminski J., Barnitz R.A., Kurachi M., Gerdemann U., Yates K.B., Tsao H.W., Godec J., LaFleur M.W., Brown F.D. (2016). The epigenetic landscape of T cell exhaustion. Science.

[bib66] Shimizu K., Sugiura D., Okazaki I.-M., Maruhashi T., Takegami Y., Cheng C., Ozaki S., Okazaki T. (2020). PD-1 Imposes Qualitative Control of Cellular Transcriptomes in Response to T Cell Activation. Mol. Cell.

[bib67] Singer M., Wang C., Cong L., Marjanovic N.D., Kowalczyk M.S., Zhang H., Nyman J., Sakuishi K., Kurtulus S., Gennert D. (2016). A Distinct Gene Module for Dysfunction Uncoupled from Activation in Tumor-Infiltrating T Cells. Cell.

[bib68] Stephen T.L., Payne K.K., Chaurio R.A., Allegrezza M.J., Zhu H., Perez-Sanz J., Perales-Puchalt A., Nguyen J.M., Vara-Ailor A.E., Eruslanov E.B. (2017). SATB1 Expression Governs Epigenetic Repression of PD-1 in Tumor-Reactive T Cells. Immunity.

[bib69] Sundstedt A., O’Neill E.J., Nicolson K.S., Wraith D.C. (2003). Role for IL-10 in suppression mediated by peptide-induced regulatory T cells in vivo. J. Immunol..

[bib70] Tang R., Langdon W.Y., Zhang J. (2019). Regulation of immune responses by E3 ubiquitin ligase Cbl-b. Cell. Immunol..

[bib71] Trapnell C., Pachter L., Salzberg S.L. (2009). TopHat: discovering splice junctions with RNA-Seq. Bioinformatics.

[bib72] Trinchieri G. (2007). Interleukin-10 production by effector T cells: Th1 cells show self control. J. Exp. Med..

[bib73] Wherry E.J., Kurachi M. (2015). Molecular and cellular insights into T cell exhaustion. Nat. Rev. Immunol..

[bib74] Wherry E.J., Ha S.J., Kaech S.M., Haining W.N., Sarkar S., Kalia V., Subramaniam S., Blattman J.N., Barber D.L., Ahmed R. (2007). Molecular signature of CD8+ T cell exhaustion during chronic viral infection. Immunity.

[bib75] Wraith D. (2016). Autoimmunity: Antigen-specific immunotherapy. Nature.

[bib76] Xu J., Yang Y., Qiu G., Lal G., Wu Z., Levy D.E., Ochando J.C., Bromberg J.S., Ding Y. (2009). c-Maf regulates IL-10 expression during Th17 polarization. J. Immunol..

[bib77] Yukawa M., Jagannathan S., Vallabh S., Kartashov A.V., Chen X., Weirauch M.T., Barski A. (2020). AP-1 activity induced by co-stimulation is required for chromatin opening during T cell activation. J. Exp. Med..

[bib78] Zhang J., Bárdos T., Li D., Gál I., Vermes C., Xu J., Mikecz K., Finnegan A., Lipkowitz S., Glant T.T. (2002). Cutting edge: regulation of T cell activation threshold by CD28 costimulation through targeting Cbl-b for ubiquitination. J. Immunol..

[bib79] Zhang Y., Liu T., Meyer C.A., Eeckhoute J., Johnson D.S., Bernstein B.E., Nusbaum C., Myers R.M., Brown M., Li W., Liu X.S. (2008). Model-based analysis of ChIP-Seq (MACS). Genome Biol..

[bib80] Zhu C., Sakuishi K., Xiao S., Sun Z., Zaghouani S., Gu G., Wang C., Tan D.J., Wu C., Rangachari M. (2015). An IL-27/NFIL3 signalling axis drives Tim-3 and IL-10 expression and T-cell dysfunction. Nat. Commun..

[bib81] Zhu L., Kong Y., Zhang J., Claxton D.F., Ehmann W.C., Rybka W.B., Palmisiano N.D., Wang M., Jia B., Bayerl M. (2017). Blimp-1 impairs T cell function via upregulation of TIGIT and PD-1 in patients with acute myeloid leukemia. J. Hematol. Oncol..

