# Supplementary material for: Chromatin Priming Renders T Cell Tolerance-Associated Genes Sensitive to Activation below the Signaling Threshold for Immune Response Genes
Source: Cell Rep. 2020 Jun 9;31(10):107748. doi: 10.1016/j.celrep.2020.107748 (PMC7296351; doi:10.1016/j.celrep.2020.107748)
Supplement: Document S1. Figures S1–S7 and Table S6 [file mmc1.pdf]

Cell Reports, Volume *31*

Supplemental Information

Chromatin Priming Renders T Cell Tolerance-Associated Genes Sensitive to Activation below the Signaling Threshold for Immune Response Genes

Sarah L. Bevington, Sky T.H. Ng, Graham J. Britton, Peter Keane, David C. Wraith, and Peter N. Cockerill

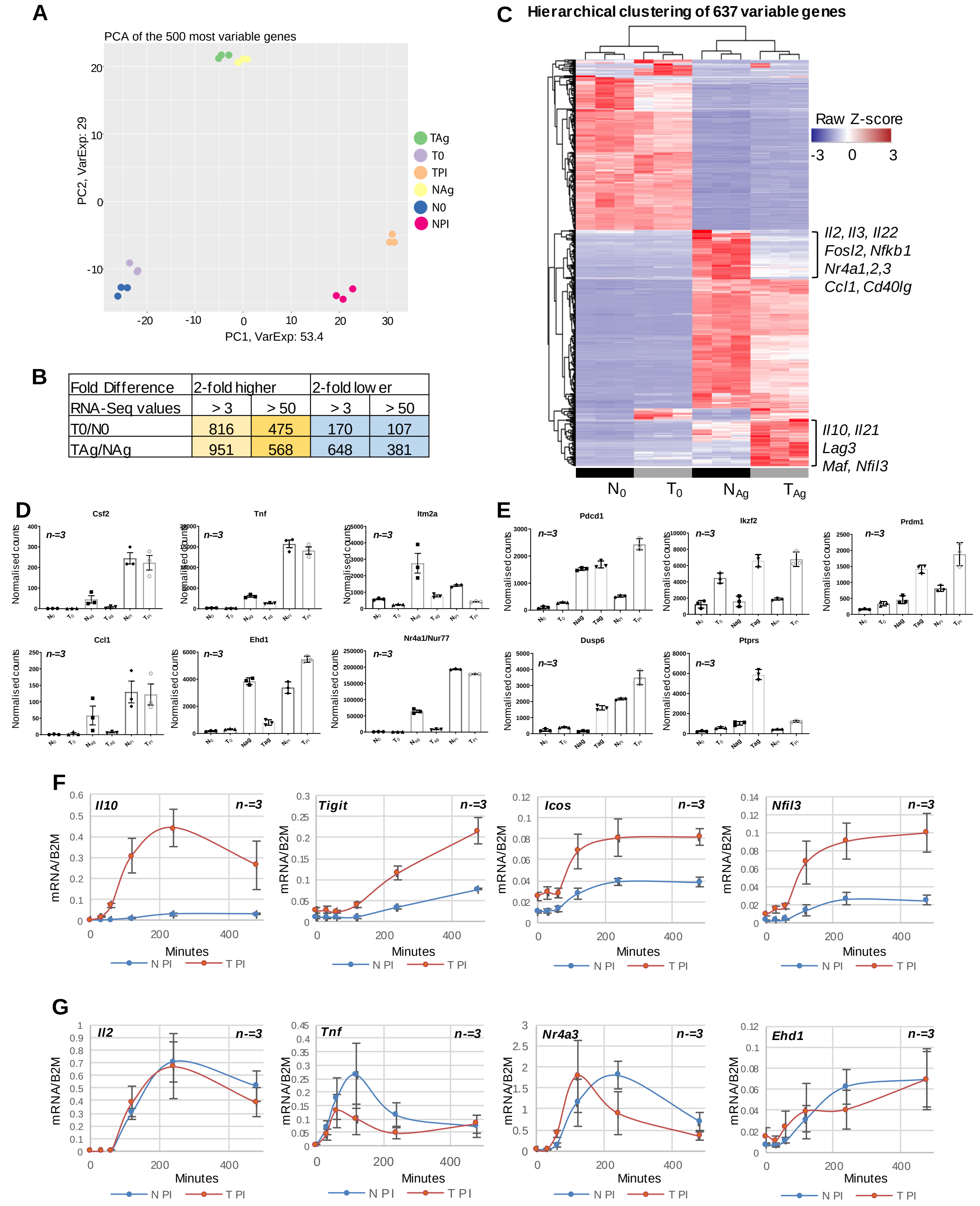

Fold Difference	2-fold higher		2-fold lower	
RNA-Seq values	> 3	> 50	> 3	> 50
T0/N0	816	475	170	107
TAg/NAg	951	568	648	381

Figure S1.

Figure S1. Gene expression analyses comparing tolerized T cells with naïve T cells.

Related to Figure 1.

(A) Principle component analysis of the RNA-Seq data sets for the most variable genes. Data was taken from 3 biological replicates.

(B) Subsets of RNA-Seq data showing numbers of genes that are at least two-fold upregulated or down-regulated in tolerized T cells.

(C) Hierarchical clustering of RNA-Seq data for N_0, T_0, N_{Ag} and T_{Ag} for 637 genes where at least one of the T_0:N_0, T_{Ag}:N_{Ag}, T_{Ag}:T_0, or N_{Ag}:N_0 ratios vary by at least 10-fold, and one value is greater than 50 for each pair. RNA-Seq data was taken from three biological replicates.

(D,E) Normalized average counts taken from RNA-Seq data of three biological replicates for immune response genes (D) and the tolerance associated genes (E).

(F,G) Inducible mRNA expression levels of immunomodulatory (F) and immune response genes (G) in Tg4 CD4 naïve and tolerant cells treated *in vitro* with PI (N_{PI}, T_{PI}) for the times indicated. mRNA levels were normalized relative to beta-2 microglobulin (B2M) expression. Standard deviation is shown for 3 biological replicates.

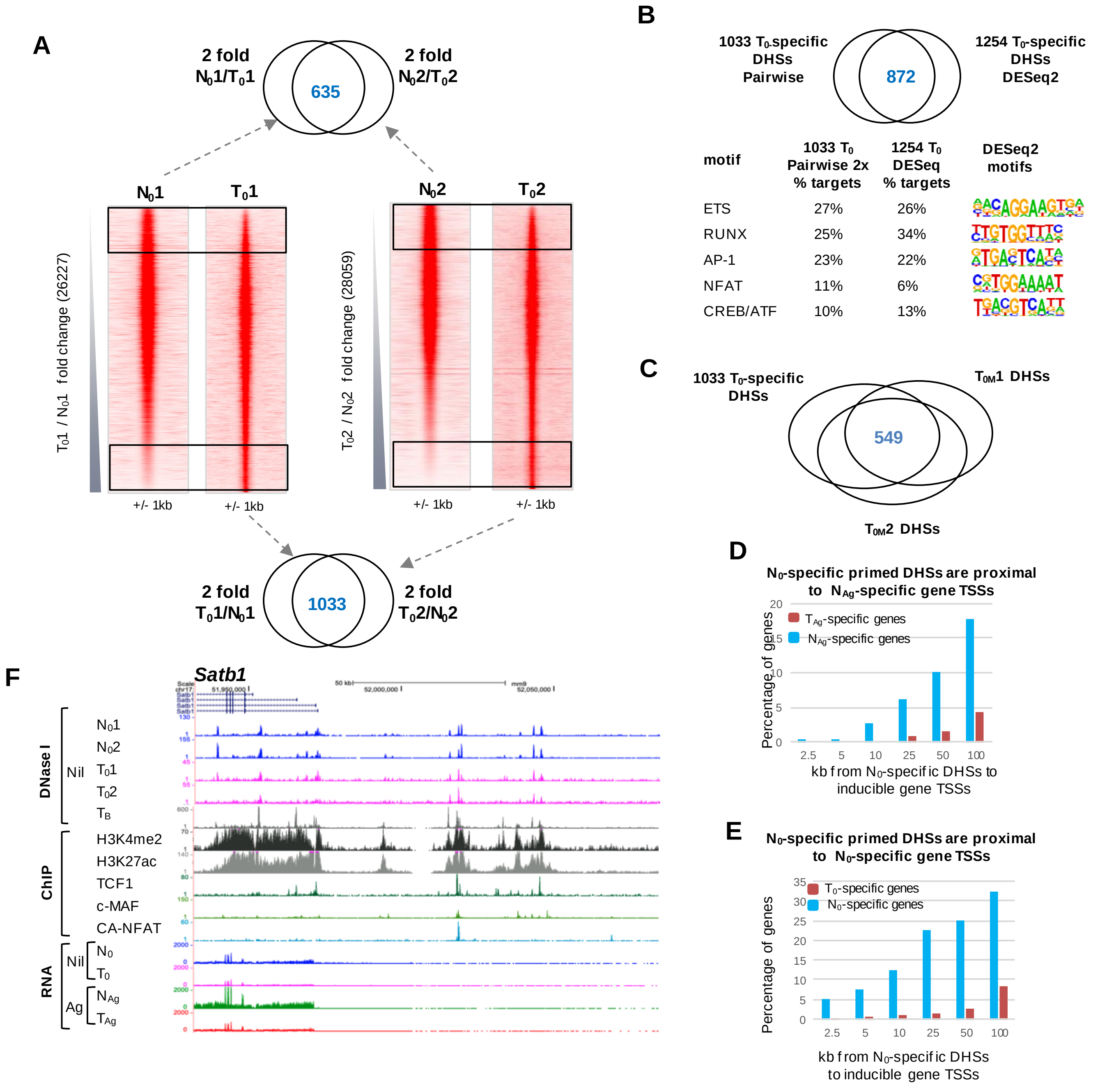

Figure S2. DNase-Seq analyses of open chromatin in naïve and tolerized T cells. Related to Figure 2.

(A) DNase-Seq tag density plots showing all 26,227 peaks detected in replicate 1 of $T_0$1 and $N_0$1 (left) and 28,059 peaks in $T_0$2 and $N_0$2 (right), ordered by increasing fold change of sequence tag count for T_0 compared to N_0. Venn diagrams s the overlap of the 2-fold specific peaks from each set of replicates for T_0/N_0 (lower) to give 1033 T_0 specific DHSs and N_0/T_0 (upper) to give 635 N_0 specific peaks.

(B) Upper. Venn diagram depicting the overlap of the 1033 2-fold T_0 specific peaks determined by the pairwise comparison with the 1254 2-fold T_0 specific peaks determined by DESeq2. Lower. HOMER *de novo* identification of TF motifs in the 1033 and 1254 peaks.

(C) Overlap of the 1033 T_0 specific peaks with the peaks detected in T_{0M}1 and T_{0M}2.

(D,E) Bar graphs showing the percentage of genes which are preferentially induced in T_{Ag} or N_{Ag} (D) and T_0 or N_0 (E) which are found within 100 kb of the 635 N_0 specific DHSs.

(F) UCSC genome browser tracks for *Satb1* showing DNase-Seq, ChIP-seq and RNA-seq data as for Figure 2G.

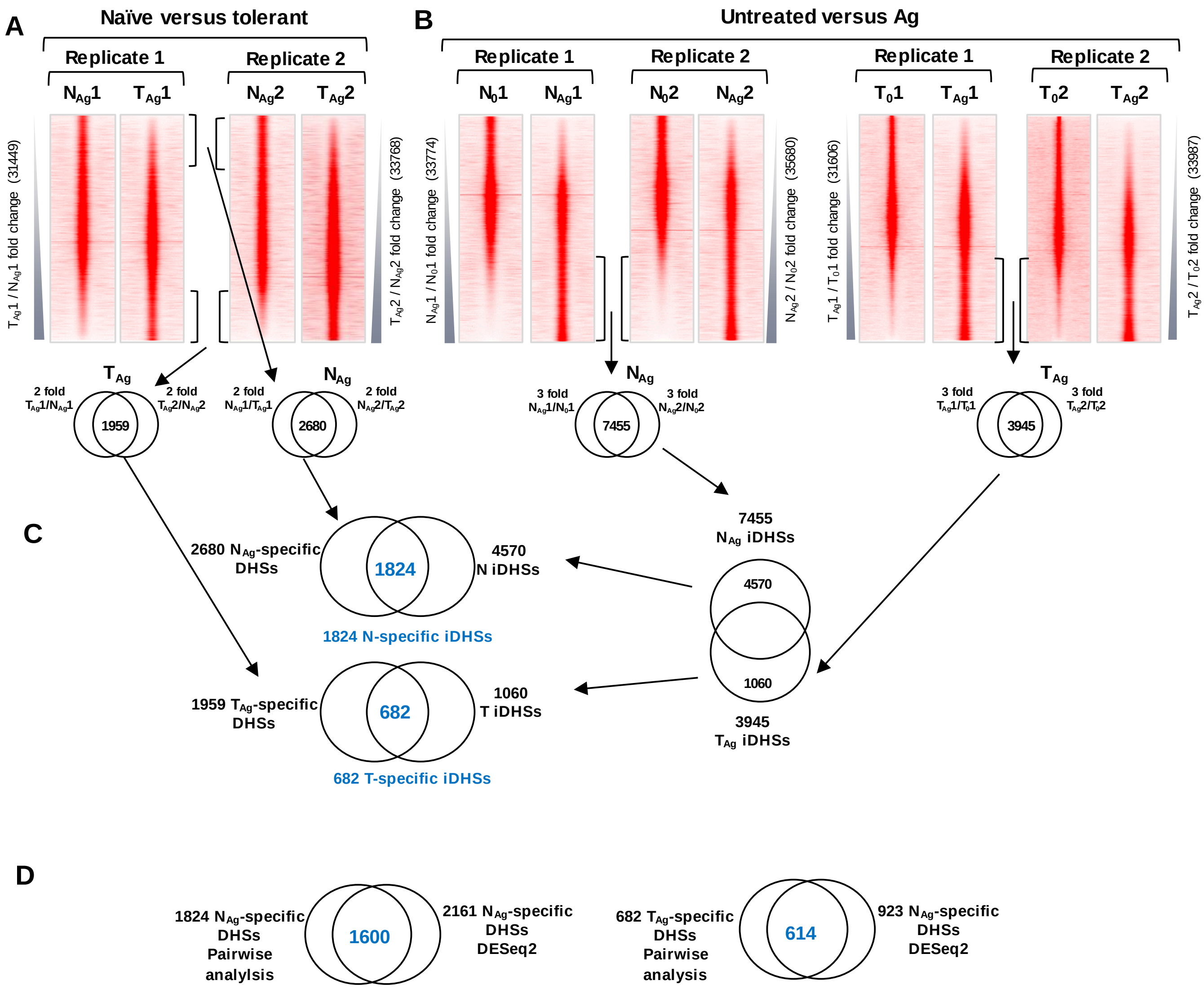

Figure S3. DNase-Seq analyses of *in vivo*-activated naïve and tolerized T cells. Related to Figure 3.

(A) DNase-Seq tag density plots showing all peaks in replicates 1 and 2 of N_{Ag} and T_{Ag} ordered by increasing fold change of sequence tag count for T_{Ag} compared to N_{Ag}. Venn diagrams show the overlap of peaks which are 2 fold enriched in the replicate T_{Ag} samples (left) and N_{Ag} samples (right).

(B) DNase-Seq tag density plots showing all peaks in replicates 1 and 2 of N_0 and N_{Ag} (left) and T_0 and T_{Ag} (right) ordered by increasing fold change of sequence tag count for Ag compared to 0. Venn diagrams show the overlap of peaks which are 3 fold enriched in the replicate N_{Ag} samples (left) and T_{Ag} samples (right).

(C) The right hand Venn diagram overlaps the >3 fold inducible DHSs present in both replicates of the naïve (7455) and tolerant (3945) to give the unique inducible DHSs for each condition (4570 N_{Ag}-iDHSs and 1060 T_{Ag}-iDHSs). The right hand Venn diagrams overlap the >2 fold enriched DHSs in the Ag-treated samples (T_{Ag} or N_{Ag} specific) with the unique iDHSs defined on the left to give the 682 Tolerant-specific iDHSs and the 1824 Naive-specific iDHSs.

(D) Overlap of the 1824 N_{Ag} specific iDHSs determined by the pairwise comparison with the 2161 N_{Ag} specific peaks determined by DESeq2 (left) and the 682 T_{Ag} specific iDHSs determined by the pairwise comparison with the 923 T_{Ag} specific peaks determined by DESeq2 (right).

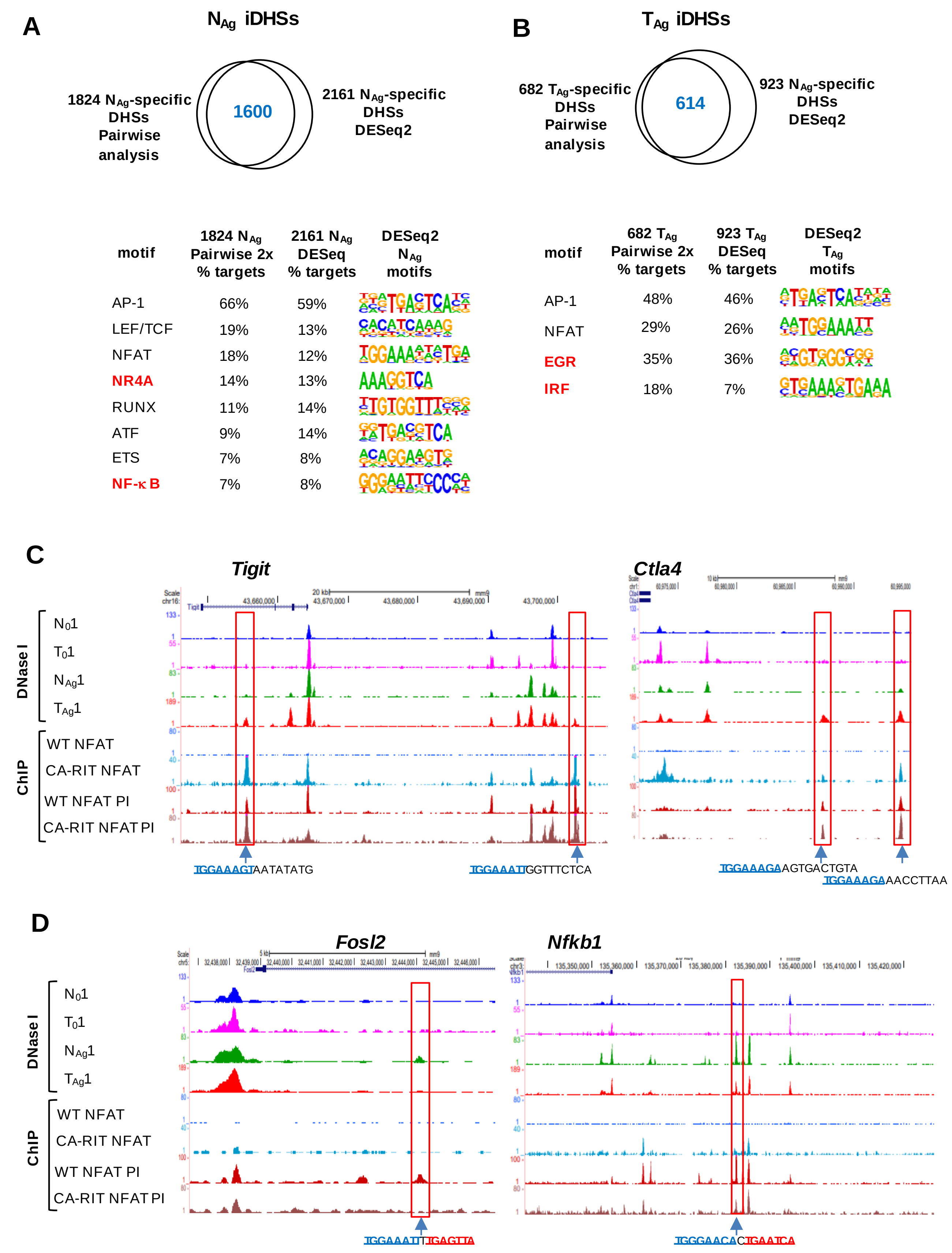

Figure S4. Tolerance-associated genes can bind NFAT independently of AP-1. Related to Figure 4.

(A,B) Homer *de novo* identification of enriched TF motifs in the 1824 N_{Ag} pairwise derived targets compared to the 2161 N_{Ag} DESeq2 derived targets (A) and the 682 T_{Ag} pairwise derived targets compared to the 923 T_{Ag} DESeq2 derived targets.

(B,C) UCSC genome browser tracks showing DNase-Seq data from resting cells (N_0 and T_0) and antigen-stimulated cells (N_{Ag} and T_{Ag}) alongside NFAT1 ChIP-seq data from CD8 WT cells +/- PI and CA-RIT-NFAT1 cells +/- PI (Martinez et al., 2015).

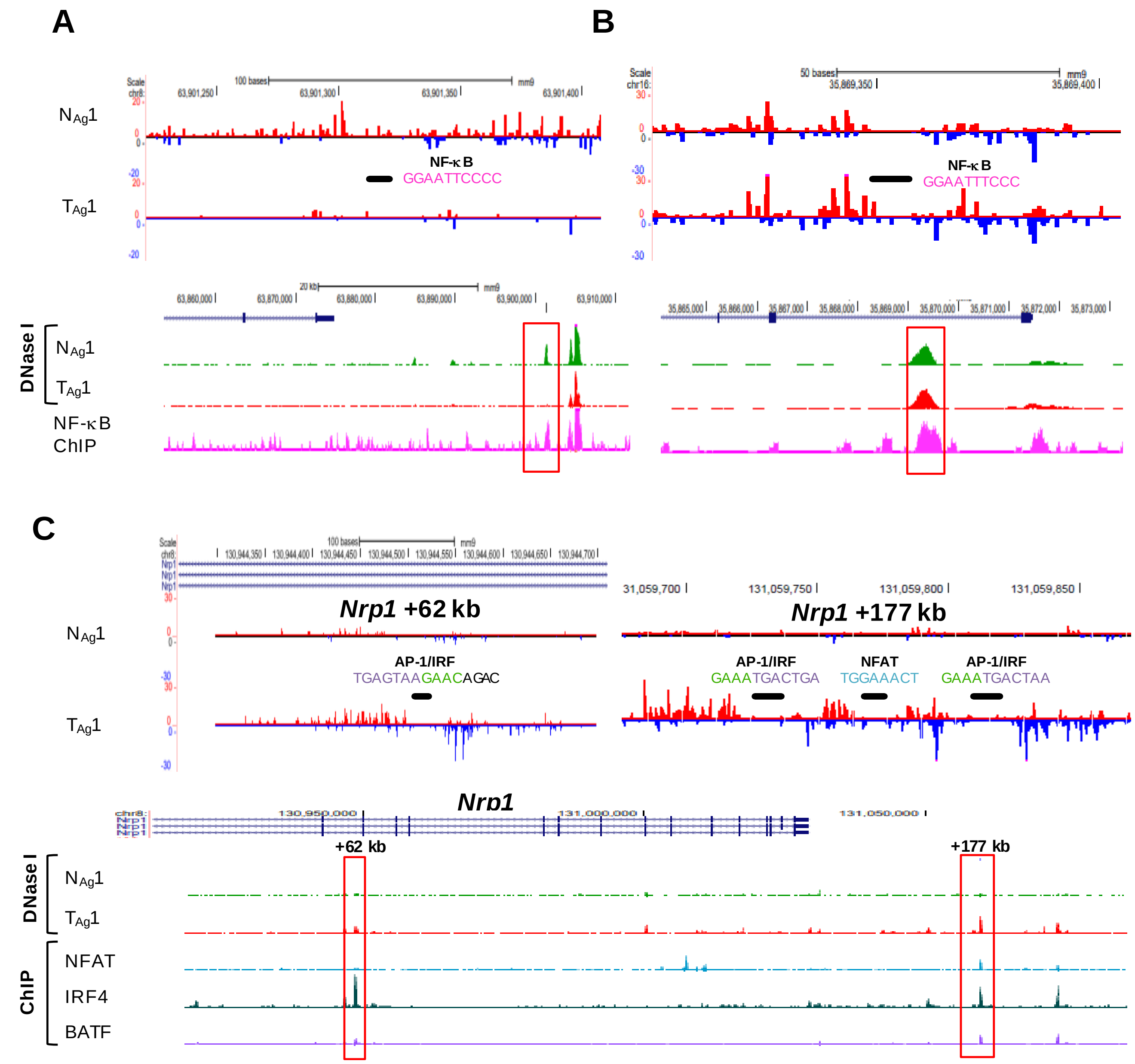

Figure S5. Examples of TF occupancy in naïve and tolerized T cells. Related to Figure 5.

(A,B) Examples of Wellington digital footprinting of DNase-Seq data showing protection of an NF-κB site at a N_{Ag}-specific iDHSs (A) and at a shared iDHSs (B) DNase-Seq data is shown for N_{Ag} and T_{Ag} and ChIP-seq data for NF-κB (Oh et al., 2017).

(C) Examples of Wellington digital footprinting of DNase-Seq data showing protection of NFAT and composite AP-1/IRF motifs DNase-Seq data is shown for N_{Ag} and T_{Ag} and ChIP-seq data for NFAT1 (Martinez et al., 2015), IRF4 and BATF (Li et al., 2012)

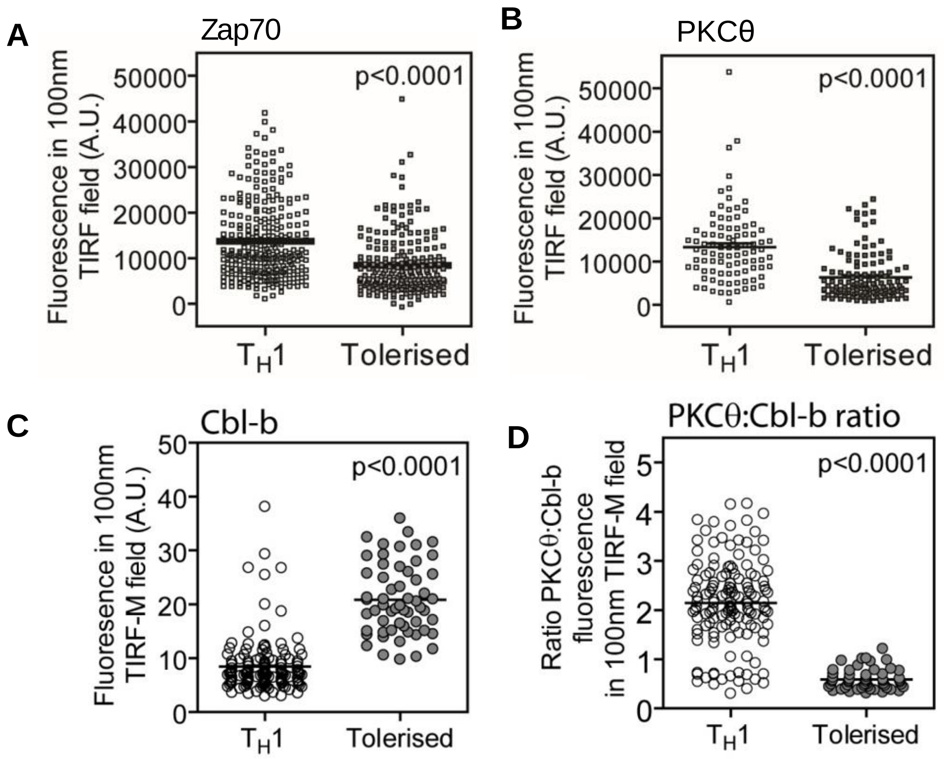

Figure S6. TIRF microscopy of protein enrichment at the tolerant T cell immunological synapse. Related to Figure 6. T_H1 and tolerant T cells were left standing on anti-CD3/CD28 coated slides for 8 minutes, labelled with fluorescent antibodies and imaged by total internal fluorescence (TIRF) microscopy. The enrichment of each protein at the T cell-slide interface was assessed by measuring the relative fluorescence in the TIRF-M imaging field.

(A-C) Accumulated TIRF data for a minimum of 30 cells for Zap70, PKCθ and Cbl-b in T_H1 cells and T cells tolerized by intranasal peptides. Each point in the plot shows data from an individual cell. Shown here are representative data from one of 4 replica experiments. Bars indicate the mean and standard error, with p values calculated by unpaired Student's t-test.

(D) Ratios of signals for PKCθ relative to Cbl-b for data depicted in B and C.

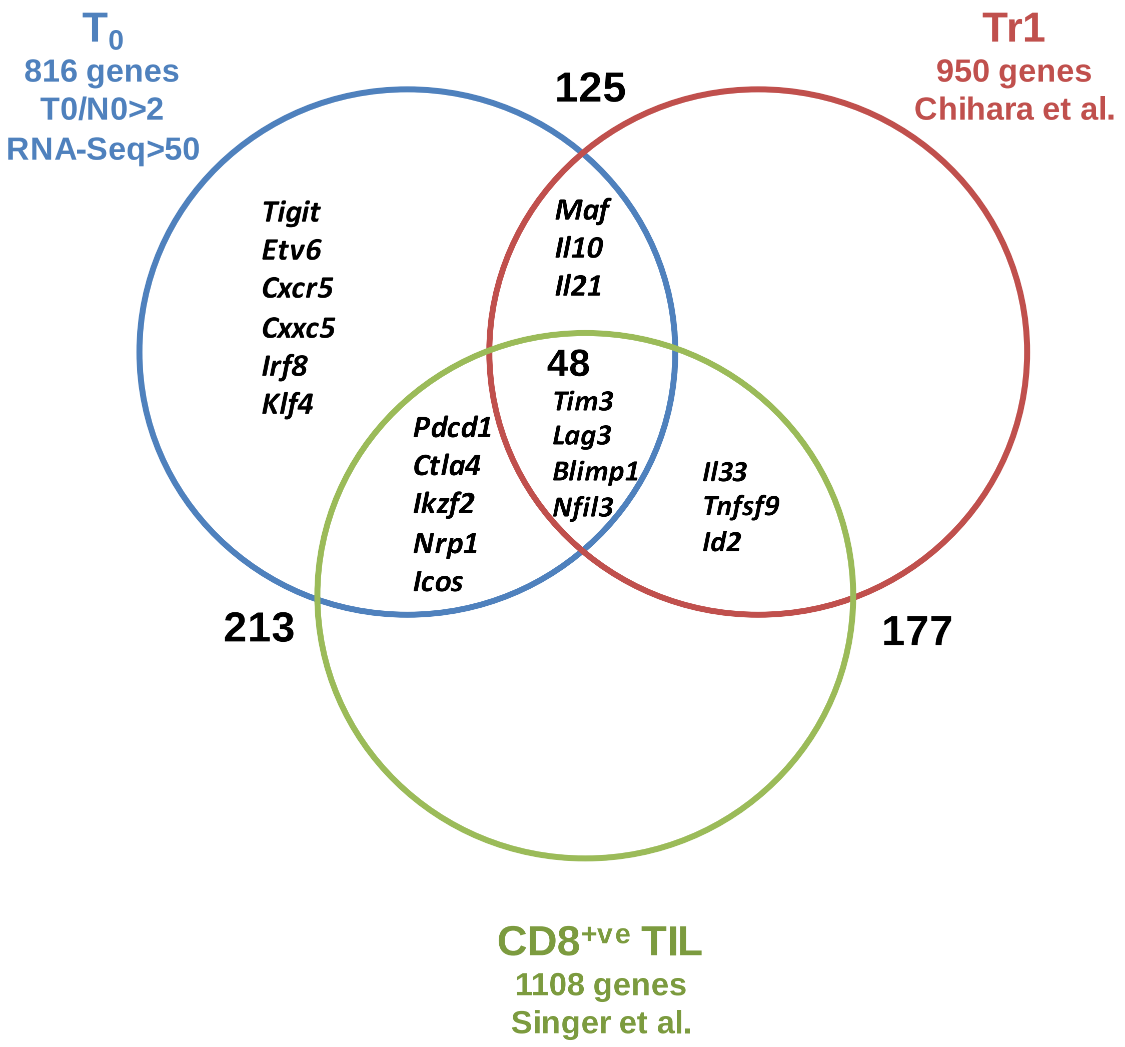

Figure S7. Venn diagram showing the overlaps in gene expression between tolerized cells, Tr1 cells and TILs. Related to Figure 1 and Figure 7.

Comparison of the 816 genes listed in Supplemental table 2 which are upregulated two-fold in T_0 compared to N_0, with genes defined as being upregulated in $CD4^{+ve}$ Tr1 cells (Chihara et al., 2018) and genes upregulated in $CD8^{+ve}$ TILs (Singer et al., 2016). A subset of genes with regulatory potential are highlighted.

**Gene**	**Forward Primer**	**Reverse Primer**
B2m	5’-TTCTGGTGCTTGTCTCACTG	5’-CAGTATGTTCGGCTTCCCATTC
Il2	5’-GATGAACTTGGACCTCTGCG	5’-CATCATCGAATTGGCACTCA
Il10	5’- CCTGGGTGAGAAGCTGAAGACC	5’-CTTCACCTGCTCCACTGCCTTG
Tigit	5’-GCAAATGAGTCCCAGCACAG	5’-GGGGAGAATATTCCTGAAGGTCC
Nr4a3	5’-CAGTGTCGGGATGGTAAGGAA	5’-CAGACGACCTCTCCTCCCTTT
Ehd1	5’- CAGGAAGCTCAATGACCTCATCAAGC	5’-GGCATCTCCTTCTTGAGGGAGC
Icos	5’-GCAGCCTGTCCATTTTTGACCCAC	5’-AGCTTCAGCTGGCAGCAGAGC
Nfil3	5’-GAACTCTGCCTTAGCTGAGGT	5’-ATTCCCGTTTTCTCCGACACG
Tnf	5’-CACGTCGTAGCAAACCACCAAGTGGA	5’-TGGGAGTAGACAAGGTACAACC

Table S6 (relates to STAR Methods resource table)

Primer sequences for real-time qPCR.
